# Digital embryos: a novel technical approach to investigate perceptual categorization in pigeons (*Columba livia*) using machine learning

**DOI:** 10.1007/s10071-021-01594-1

**Published:** 2022-01-06

**Authors:** Roland Pusch, Julian Packheiser, Charlotte Koenen, Fabrizio Iovine, Onur Güntürkün

**Affiliations:** 1 Department of Biopsychology, Faculty of Psychology, Ruhr University Bochum, Universitätsstraße 150, 44780 Bochum, Germany; 2 The Social Brain Lab, Netherlands Institute for Neuroscience, Meibergdreef 47, 1105 BA Amsterdam, Netherlands

**Keywords:** Virtual phylogenesis, Visual system, Avian, Learning, Common elements

## Abstract

Pigeons are classic model animals to study perceptual category learning. To achieve a deeper understanding of the cognitive mechanisms of categorization, a careful consideration of the employed stimulus material and a thorough analysis of the choice behavior is mandatory. In the present study, we combined the use of “virtual phylogenesis”, an evolutionary algorithm to generate artificial yet naturalistic stimuli termed digital embryos and a machine learning approach on the pigeons’ pecking responses to gain insight into the underlying categorization strategies of the animals. In a forced-choice procedure, pigeons learned to categorize these stimuli and transferred their knowledge successfully to novel exemplars. We used peck tracking to identify where on the stimulus the animals pecked and further investigated whether this behavior was indicative of the pigeon’s choice. Going beyond the classical analysis of the binary choice, we were able to predict the presented stimulus class based on pecking location using a k-nearest neighbor classifier, indicating that pecks are related to features of interest. By analyzing error trials with this approach, we further identified potential strategies of the pigeons to discriminate between stimulus classes. These strategies remained stable during category transfer, but differed between individuals indicating that categorization learning is not limited to a single learning strategy.

**Supplementary Information:**

The online version contains supplementary material available at 10.1007/s10071-021-01594-1.

## Introduction

Categorization can be defined as the ability to classify stimuli by responding equivalently to members of the same class, unequally to members of different classes, and to transfer this knowledge to novel instances of these categories (Cohen and Lefebvre 2017; Keller and Schoenfeld 1950). In comparative research, pigeons are a valuable model organism to study category learning as they have been shown to be able to categorize extremely complex naturalistic visual stimuli. For examples, they can reliably categorize photographs with humans vs. those without (Aust and Huber 2001, 2002; Herrnstein and Loveland 1964), words vs. nonwords (Scarf et al. 2016), benign vs. cancerous histological samples (Levenson et al. 2015), and pictures of healthy and diseased heart muscle (Navarro et al. 2020). However, especially for complex naturalistic stimuli, the question remains on which stimulus features this well-documented categorization ability is based. In the present study, we use artificial naturalistic stimuli and analyze the pigeon’s pecking behavior using machine learning to gain insights into the process of perceptual categorization.

**Fig. 1:**
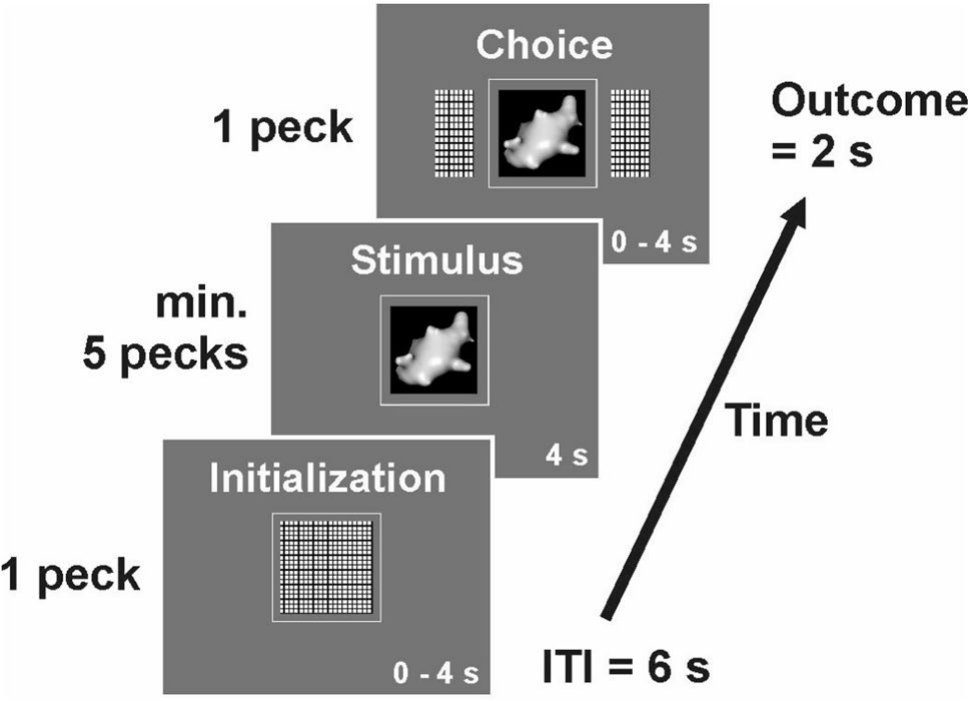
Example trial of the categorization task. Each trial began with a short high tone. First, the initialization key was presented for up to 4 s and could be terminated once pecked. Then, one digital embryo stimulus was presented (depicted is a digital embryo out of class X) and had to be pecked at least 5 times. It could not be terminated for a fixed time interval of 4 s. Subsequently, two choice keys were presented alongside the stimulus for 4 s. One peck on either key indicated the pigeon’s choice and was either immediately rewarded with 2 s access to grain or punished with lights turned off and a low punishment tone for 2 s. For half of the pigeons, the right choice key represented class X and the left choice key represented class Y (vice versa for the other half of the pigeons). The next trial followed after a 6 s inter-trial interval

Different approaches have been employed to isolate diagnostic features of stimuli that drive perceptual categorization learning. One approach controls the feature content using carefully constructed artificial stimuli to investigate categorization mechanisms (e.g. Jitsumori 1996). These kind of experiments showed that color, size, shape and their combined configural cues all seem to be behaviorally relevant (Wasserman and Biederman 2012). However, due to the narrow detail in artificial, geometric stimuli, the problem of limited generalizability to natural visual classes remains (Lazareva and Wasserman, 2017). Other attempts use complex natural stimuli (e.g. Fersen and Lea 1990) at the expense of being able to clearly demonstrate the exact features the animals were responding to (Aust and Huber 2001). The reason is possibly that in natural categories different reward predicting features co-occur and many of such features may contribute to the class assignment. Thus, the precise identification of pictorial features exerting control over behavior is astonishingly difficult (e.g., Aust and Huber 2002). Here, we use “digital embryos” to study perceptual categorization (see Fig. 1). Digital embryos are stimuli developed from a single parent object that was successively morphed through iterations into their final shapes. These stimuli have been utilized as a versatile tool for categorization learning research (Hauffen et al. 2012; Hegdé et al. 2008; Kromrey et al. 2010; Tabrik et al. 2021). The growth of these similar but distinct objects is realized by an evolutionary algorithm called “virtual phylogenesis” (VP). VP can create any number of classes and class members with varying degrees of complexity and difficulty in discrimination. Such biologically inspired algorithms have proven to be useful tools in comparative cognition research, allowing the analysis of animal behavior beyond the binary choice of correct or incorrect responses (Bond and Kamil 2006; Cook and Qadri 2013). Thus, the virtual evolution of digital embryos mimics natural objects that possess features that are readily controlled by the experimenter.

To identify informative diagnostic variables of a specific category that pigeons might utilize in a categorization task we used peck tracking (Allan 1993; Dittrich et al. 2010; Jitsumori and Yoshihara 1997). Dittrich et al. (2010) could show that pigeons working on a people present/people absent categorization task preferentially pecked on the heads of the depicted persons and were subsequently impaired in their performance when they had to run the task with the heads of the humans removed from the photographs. Similarly, Castro and Wasserman (2014, 2017) demonstrated that pigeons track category-relevant features and respond less to details that only weakly coincide with the presence of the relevant stimulus.

By combining VP evolved stimuli and peck tracking, the central purpose of our study is the detailed analysis of individual pecking behavior in response to digital embryo stimuli using machine learning. We trained a classifier to identify the relationship between a behavioral outcome, i.e. either correct or incorrect choice, and the underlying pecking pattern. By training the classifier on correct trials, we aim to identify whether pecking locations were predictive of the presented category. Training the classifier on incorrect trials further gives insights into the underlying learning strategy of individual animals.

In our experiments, we trained pigeons to distinguish between two different classes of digital embryos in a single interval forced-choice task. First, the animals went through a category learning procedure and second, conducted a transfer test with new stimuli of the same class. Peck tracking using touch-screen technology allowed us to gain insight into local attentional mechanisms and categorization strategies. Our hypotheses of the following experiments were: (1) pigeons, in line with humans and monkeys, can discriminate between the two classes of digital embryos. (2) Categorical information can be transferred to new exemplars of a given category. This can be viewed as a proof of open-ended categorization, a level beyond rote categorization (Herrnstein 1990). (3) Using machine learning, we can successfully predict the presented stimulus class only based on pecking location ultimately providing insight into the features used for discrimination as well as the underlying strategy of each individual animal.

## Methods

### Subjects

Eight unsexed adult homing pigeons (*Columba livia*) obtained from local breeders served as subjects. The pigeons were housed in individual wire-mesh cages with a 12-h light–dark cycle beginning at 8:00 a.m. They had free access to water and were food deprived, maintained at approximately 80–90% of their free feeding body weight and fed accordingly with a mixture of different grains. The subjects were treated in accordance with the German guidelines for the care and use of animals in science. All procedures were approved by a national ethics committee of the State of North Rhine-Westphalia, Germany. All experimental conduct was in agreement with the European Communities Council Directive 86/609/EEC concerning the care and use of animals for experimental purposes.

### Apparatus

The experiment was conducted in a custom-built operant chamber (35 × 35 × 35 cm) with a touch-screen monitor (model ET1515L with APR technology, Elo Touch Solutions Inc., Milpitas, CA, USA), on which three horizontally aligned areas were defined as pecking keys (cf. Fig. 1; central pecking key 5 × 5 cm, lateral pecking keys 3.5 × 5 cm with a 1 cm gap in between pecking keys; 17 cm above the chamber floor).

The touch screen was used to present the stimuli, record pecks and location coordinates for each peck. The stimuli measured 4 × 4 cm and were presented within the central pecking key, surrounded by a 0.5 cm gray border. The lateral pecking keys measured 2.5 × 4 cm, surrounded by a 0.5 cm gray border that blended with the gray background of the touch-screen. In both cases, pecking this gray border was registered as a valid peck. This buffer was included because there is no distinct physical pecking key on touch screens and pigeons sometimes peck on the edge of a stimulus display. The grey border enables these pecks to be included. A centrally located food hopper delivered mixed grains as reward. Experimental hardware was controlled with custom-written Matlab code using the Biopsychology-Toolbox (Rose et al. 2008).

### Stimuli

**Fig. 2:**
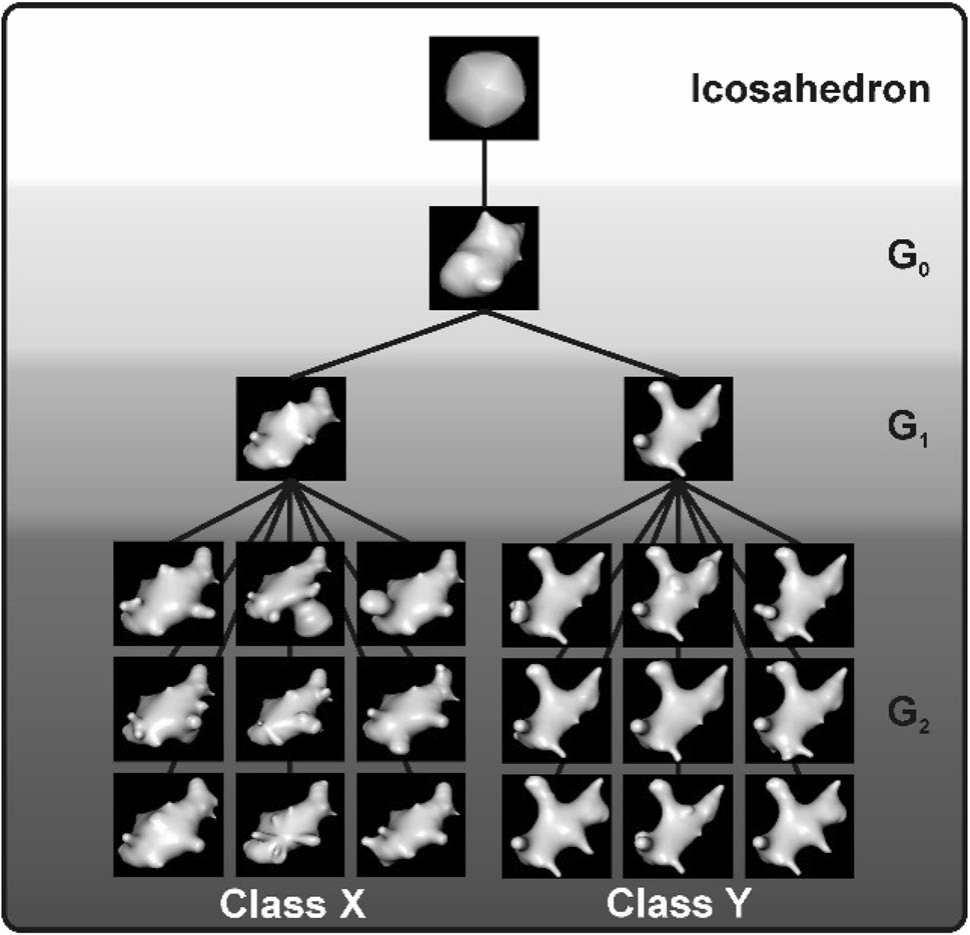
Digital embryo classes. Digital embryos were created with a process called “virtual phylogenesis” that mimics biological evolution. Starting with an icosahedron, different generations (G_0_–G_2_) are created, resulting in embryo classes X and Y

Two classes of digital embryos, arbitrarily termed X and Y, were generated using the software Digital Embryo Workshop (Brady and Kersten 2003; Hauffen et al. 2012). These classes were created using a process called “virtual phylogenesis” an algorithm that mimics biological evolution by creating multiple virtual taxonomic classes of 3D objects that have been termed “digital embryos”. This process starts with an icosahedron (a polyhedron with 20 faces) as the parent object. Over several virtual generations multiple separate classes “evolve”, each of which shares identifying within-class characteristics. 50 digital embryos of each class were initially created. Using a pixelwise correlation between the stimuli, we selected 30 stimuli per class with the lowest correlation coefficients to members of the other class resulting in clearly distinguishable stimulus classes. Our selection of exemplars ensured that the created stimuli could not be categorized based on their size nor based on the amount of the surrounding black area (see supplementary Fig. 1). Example embryos from different generations including class X and Y are depicted in Fig. 2 and full sets of stimuli are depicted in supplementary Figs. 2 and 3. In our study, class X and class Y consisted of 30 members each, 20 of which were randomly chosen for category training and 10 used for transfer tests.

### Behavioral task

Pigeons were trained in daily sessions (5 days a week) on a single interval forced-choice task (Fig. 1). The start of each trial was indicated by a short high-frequency tone (500 Hz; Logitech, Model S-120). Pigeons first had to peck at least once on an initialization key in a 4 s time window to commence the trial. Then, one digital embryo stimulus was presented for a fixed time interval of 4 s. During this period, the pigeons were required to peck at least five times on the stimulus. If they did not, the trial was aborted. If the bird had pecked five times during the 4 s interval, two choice keys were presented alongside the stimulus. Subsequently, pigeons had to make a choice, i.e. peck once on either the left or right choice key within a 4 s time window. The stimulus–response association was balanced across pigeons. When a choice was made, the display disappeared. For correct choices, the animals had 2 s access to food and the feeding light was turned on. For incorrect choices, the ambient chamber lights were turned off for 2 s accompanied by a low frequency tone (80 Hz). The next trial followed after a 6 s inter-trial interval (ITI). For experimental reasons, the paradigm had to be divided into two phases.

#### Embryo learning phase

During an initial training phase, pigeons learned to peck on all three pecking keys in a standard autoshaping procedure. Subsequently, they learned to peck the central key first, and then one choice key second, to obtain a food reward. During category training, all stimuli (20 class X, 20 class Y) were presented 5–10 times in a pseudo-randomized order. Eighty percent of trials were forced-choice trials, i.e. only the correct choice key was visible. The remaining 20% of the trials were free-choice trials. This means both choice keys were visible, could be pecked on, and errors could be made. Forced-choice trials were gradually reduced to zero once pigeons correctly categorized the stimuli in more than 80% of the free-choice trials in two consecutive sessions. Additionally, the probability of food reward was gradually reduced from 100 to 60% to ensure stable behavioral performance in longer sessions. The programmed level of reinforcement for the following phase was always set to 60% (see supplementary table 1).

#### Transfer test phase

In the transfer test phase, pigeons’ choices to transfer stimuli, i.e. new whole embryo exemplars of class X and Y and choices to known stimuli, i.e. embryo exemplars learned during training, were tested. The stimulus sequence consisted of 20 known whole embryos of each class, which were repeatedly shown 4 times (80 class X + 80 class Y = 160). These trials are labeled known-stimulus trials. Further, the sequence included the presentation of 10 new embryos (10 class X + 10 class Y = 20). These trials are labeled transfer-stimulus trials. Each individual session comprised up to two of these blocks, with a reward probability ranging between 43 and 72% (mean 57%) depending on the individual performance of the animal.

In a first transfer test phase, animals received no feedback for behavioral responses in transfer-stimulus trials, thus, reward or punishment was omitted irrespective of the animals’ responses. In this manner, transfer stimuli could be tested without reward contingencies influencing the pigeons’ decision. Unfortunately, under these conditions, four pigeons refused to peck altogether. Therefore, we repeated the transfer phase during which all pigeons received non-differential reinforcement for transfer trials, i.e. food reward irrespective of correct or incorrect choice (van Hamme et al. 1992). This motivated the animals to attend to the task without differentially reinforcing one response over another in test conditions. We will only report the results from the reinforced transfer test in the main results as the behavioral performance during the non-reinforced and reinforced transfer were virtually identical for the four pigeons who responded in both transfer tests (data from non-reinforced transfer can be found in the supplementary results).

### Data analysis

**Fig. 3:**
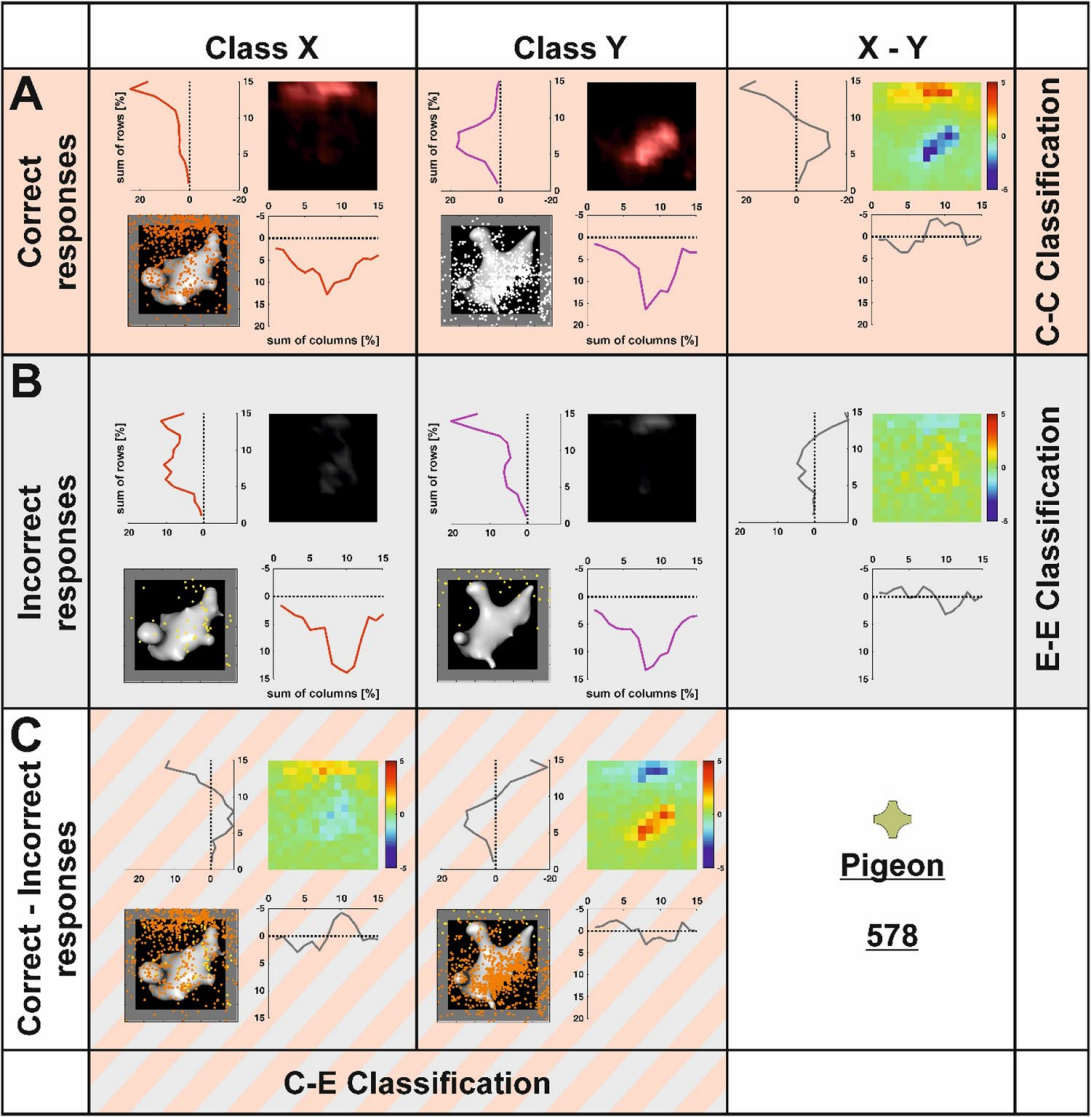
Rationale of the heatmap analysis exemplified for pigeon 578. In row A, the peck distribution for correctly responded stimulus presentations is shown separately for stimulus class X and Y. To create heatmaps, the stimulus display was divided in sections of 15 × 15 squares and pecks were counted in each section. Heatmaps were created based on relative pecks to compare individual animals. The subtraction of both heatmaps reveals the different locations of pecks for each stimulus class. The precise location of each peck is used by the classifier to separate both classes resulting in the correct–correct (CC) classification. Row B shows the peck distribution for incorrectly responded stimulus presentations. The subtraction of both heatmaps reveals the different locations of pecks for each stimulus class in error trials. The precise location of each peck is used by the classifier to separate both classes resulting in the error–error (EE) classification. Row C depicts the subtraction of correct and incorrect heatmaps for each class separately resulting in the correct–error (CE) classification. The depicted analysis is exemplary based on the responses of pigeon 578 (the symbol above the identifier number indicates this individual pigeon throughout all figures). Response profiles of each individual pigeon are given in supplementary Figs. 4–10

Pigeons’ pecking and choice behavior was stored and subsequently analyzed in MATLAB Version 2020a. We calculated the percentage of correct choices collapsed for both classes X and Y for each animal individually. This was done for known and transfer stimuli across both experimental phases. We then calculated one-sample *t* tests to identify if performance was different from chance level (50% as the choice was binary). To identify if performance on transfer trials differed from performance on known-stimuli trials, a paired *t* test was conducted. The same procedure was repeated for stimuli of class X and Y separately. Furthermore, we used precise peck tracking to gain insight into the pigeons’ center of attention. Using touch-screen technology *x*- and *y*-coordinates of each peck in the stimulus/border area were stored and analyzed for each stimulus presentation in both known- and transfer-stimulus trials. If the pigeon pecked outside the stimulus/border area, data on these pecks was discarded. Pecks on all stimuli of class X and Y, respectively, were collapsed and displayed in heat-maps separately for each pigeon to visualize each animal’s center of attention. To create heatmaps, the image was sectioned into 15 × 15 equally sized squares (Fig. 3). Each single square covered an area of 0.11 cm^2^ of the stimulus display. Pecks located in each square were summed and because the total number of pecks differed between pigeons, the relative number of pecks (relPecks = (100/allPecks) * pecks/square) is depicted in the heatmaps.

To analyze the peck distribution of pigeons, we summed the number of pecks in each square of the stimulus display. Beginning with the square in which most of the pecks were located, we calculated the cumulated sum by adding the next square in a descending order. For each square, we then computed how many pecks with regard to the total number of pecks were associated with each of the 225 squares. If the animals pecked in a focused manner, few squares would receive high values in this analysis. If the animals pecked in a more dispersed manner, the pecks would be more distributed across a larger number of squares. To quantify whether pecks in correct and error trials differed for each individual animal, we compared the cumulative distributions of relative pecks per square for both stimulus classes between correct and error trials using a Kolgomorov–Smirnov test. Since we calculated this difference for each animal individually, we corrected our significance threshold using Bonferroni’s method resulting in an adjusted *α* = 0.05/8 or *α* = 0.00625.

### Classifier analysis

To identify if peck locations were behaviorally relevant for each individual animal, we used a k-nearest neighbor (kNN) classifier algorithm to predict the presented stimulus class based on the pecking location. The fitcknn function in MATLAB was used for implementation of the algorithm. The classifier analysis was performed in known- and transfer-stimuli trials. For all correctly responded trials, we randomly sampled *n* = 250 pecking events from the behavioral session and trained the algorithm as follows: the *x-* and *y*-coordinates of each peck were associated with the associated label (0 for class X trials, 1 for class Y trials). A classification was then computed using *k* = 15 nearest neighbors according to the recommendation to use an uneven number of neighbors as well as *k* = sqrt(*n*) of training events for good classification accuracy and low computational expenses (Lall and Sharma 1996).

In a next step, 250 randomly chosen pecking events from correct trials (different from the training events) were labeled according to the kNN classifier based on their pecking location. Accuracy of the classifier was measured by quantifying the success rate of correctly classifying the presented stimulus class. Classification was performed across ten iterations each feeding random pecking events as training trials and predicting another subset of test trials to cross-validate the input and output of the algorithm and identify the variance in accuracy. To quantify if the results from the classifier were statistically significant from a chance-based estimate, we repeated ten iterations of the classifier, but fed the algorithm with shuffled input where class labels and *x*- and *y*-coordinates were permutated randomly. A dependent *t* test between the results from the empirical and shuffled data was then performed. Results for the *t* tests were corrected using the Bonferroni method since they were conducted individually for eight pigeons. The adjusted threshold for significance was therefore lowered to *α* = 0.05/8 or *α* = 0.00625. Since this analysis trained and tested the algorithm with pecking events from correct trials, we will refer to this classification as correct–correct or CC classification.

If sufficient pecking events occurred after which the animals made erroneous choices (> 500 events), we also quantified if pecking locations from correct trials were predictive of error trials to identify whether pecking in correct trials and error trials was consistent or differed from one another. For that purpose, the aforementioned analysis was repeated, but test pecks for the algorithm were chosen from erroneous trials rather than correct trials. This analysis will subsequently be referred to as correct–error or CE classification. Furthermore, if sufficient pecking events from error trials occurred, we also predicted the class label using error trials as both training and test events. Aim of this analysis was to quantify the internal consistency in pecking during error trials, i.e. if the stimulus class in error trials can be predicted based on pecking location in error trials. This analysis will be dubbed error–error or EE classification.

## Results

### Embryo learning phase

The embryo learning phase took between 29 and 49 sessions to meet criteria and move on to the next phase (for experimental details see supplementary table 1). All eight pigeons performed two consecutive sessions at 80% correct of the free-choice trials before moving to the next phase. After reaching the initial learning criterion, a step-wise reduction of forced-choice trials as well as of reward probability followed to move to the transfer test phase.

### Transfer test phase

**Fig. 4:**
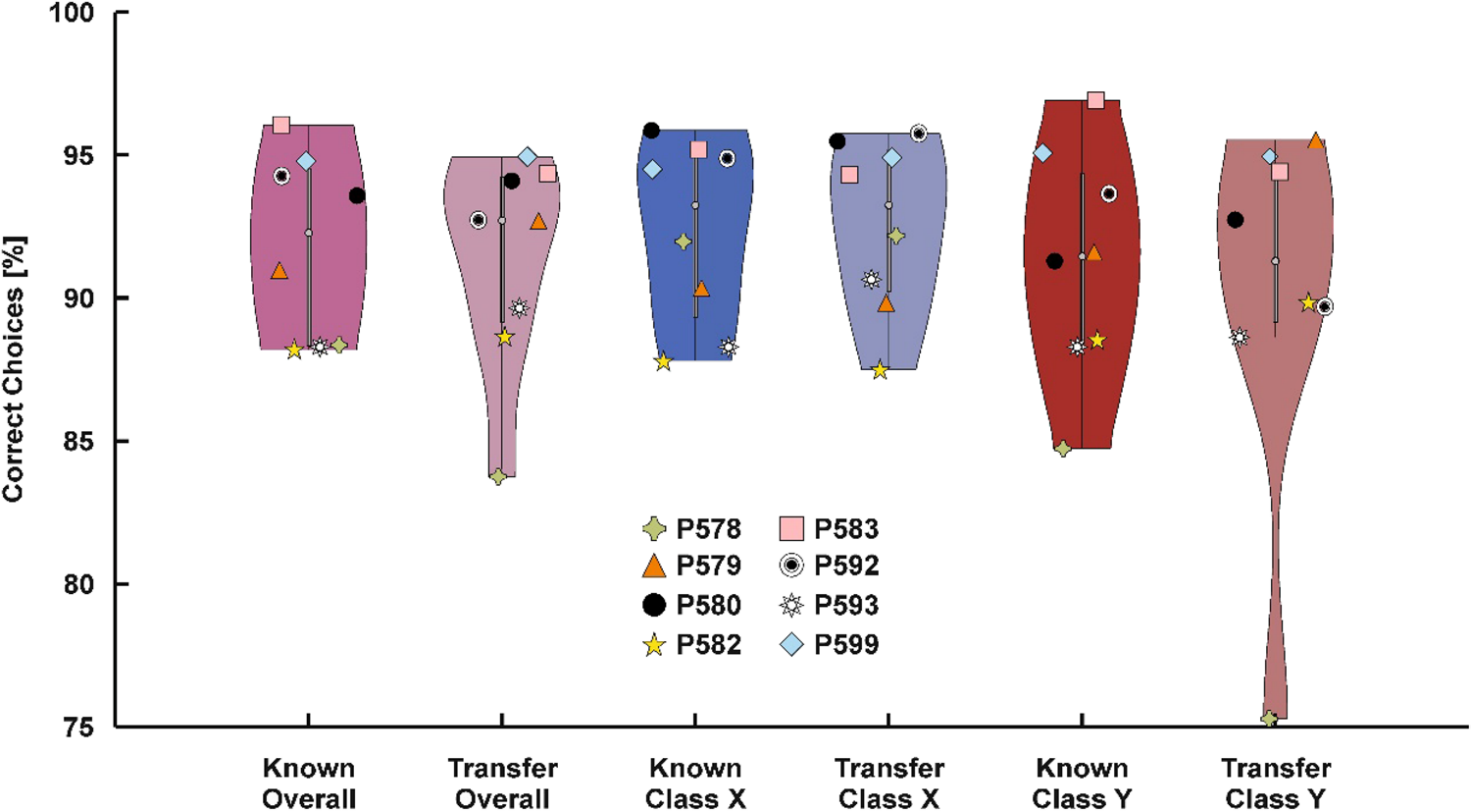
Behavioral results. Violin plot of behavioral performance for known and transfer trials overall and broken down by embryo class. Digital embryos could be categorized in each experimental condition under non-differential reward conditions. There was no difference between the performance to the known embryos and the transfer to new instances of embryo classes X and Y in any of both conditions. The symbols represent the performance of each individual pigeon and apply throughout all figures. Boxplots represent the lower quartile (Q1), the median and the upper quartile (Q3). Whiskers represent Q1 − 1.5 * interquartile range (IQR) and Q3 + 1.5 * IQR

All eight pigeons were successfully tested on known- and transfer-stimuli during the test session when transfer trials were rewarded non-differentially. Overall, performance in known-stimuli and transfer-stimuli trials was very high (Mean 91.81%, SD 3.26%; Mean 91.36%, SD 3.80%, respectively, see Fig. 4) and was significantly different from chance [*t*(7) = 4260.45, *p* < 0.001, Cohen’s *d* = 12.83; *t*(7) = 3.654.37, *p* < 0.001, Cohen’s *d* = 10.89, respectively]. There was no difference in performance between known- and transfer-stimuli trials [*t*(7) = 0.61, *p* =  > 0.250, Cohen’s *d* = 0.45]. Individually, the two stimulus classes, both class X (known-stimuli trials mean 92.89%, SD 3.23%; transfer-stimuli trials mean 92.58%, SD 3.03%) and class Y (known-stimuli trials mean 91.26%, SD 3.98%; transfer-stimuli trials mean 90.13%, SD 6.55%), showed above chance-level performance (all *p*s < 0.001). There was no difference between stimuli of class X and Y in both known- [*t*(7) = 0.97, *p* > 0.250, Cohen’s *d* = 0.32] and transfer-stimuli trials [*t*(7) = 1.02, *p* =  > 0.250, Cohen’s *d* = 0.42]. Behavioral results of the four pigeons who completed the transfer test in the absence of reward are presented in the supplementary results.

### Peck tracking

We used a k-nearest neighbor classifier to identify whether the presented stimulus class can be predicted based on the pecking location. To this end, the classifier was trained with randomly chosen pecking events across behavioral sessions in which pecking events were labeled according to the presented stimulus class in a given trial. Another subset of trials was then used to determine the predictive accuracy of the classifier based on pecking location alone (see “Methods”). This was done for each animal separately. Results of all analyses for the four pigeons that successfully performed the non-reinforced transfer are presented in the supplementary data.

**Fig. 5:**
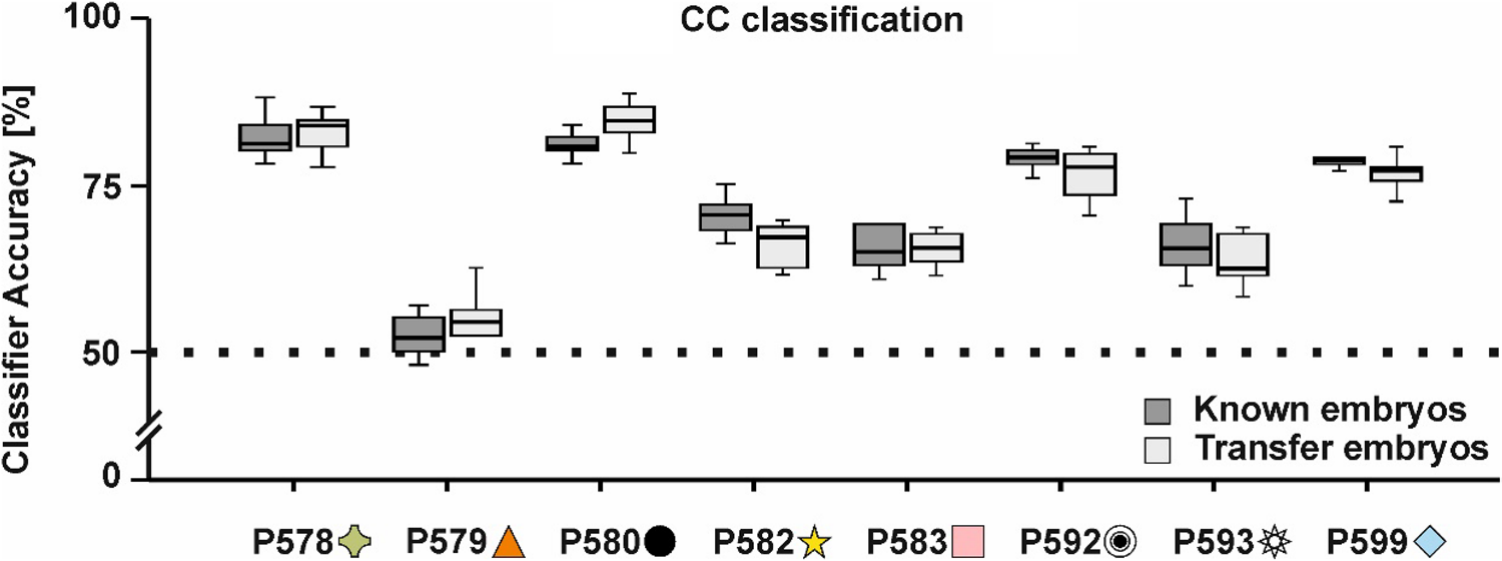
CC classification results. Digital embryos could be classified for seven out of eight animals tested. Classifier responses for the known stimuli are shown in dark gray and the classifier results for the transfer stimuli are depicted in light gray. Boxplots represent the lower quartile (Q1), the median and the upper quartile (Q3). Whiskers represent Q1 − 1.5 * IQR and Q3 + 1.5 * IQR

Our aim was to investigate whether the class label can be predicted in correct–correct or CC classification in which both training and test trials were constituted by correct trials. All eight animals could be investigated for a CC classification in known-stimuli and transfer-stimuli trials. For known-stimuli trials, we found that the classifier could significantly predict the stimulus class based on pecking location for seven out of eight pigeons (P578: 82.08%, P580: 80.84%, P582: 70.56%, P583: 64.24%, P592: 77.84%, P593: 64.64%, P599: 77.80%, all *p*s < 0.001; for the classification accuracy based on shuffled input data see supplementary Fig. 11). Only results for pigeon P579 did not differ significantly from chance [52.40%, *t*(9) = 1.22, *p* > 0.250, Cohen’s *d* = 0.29, see Fig. 5] as the animal indifferently pecked on one side of the stimulus irrespective of the presented stimulus class.

**Fig. 6:**
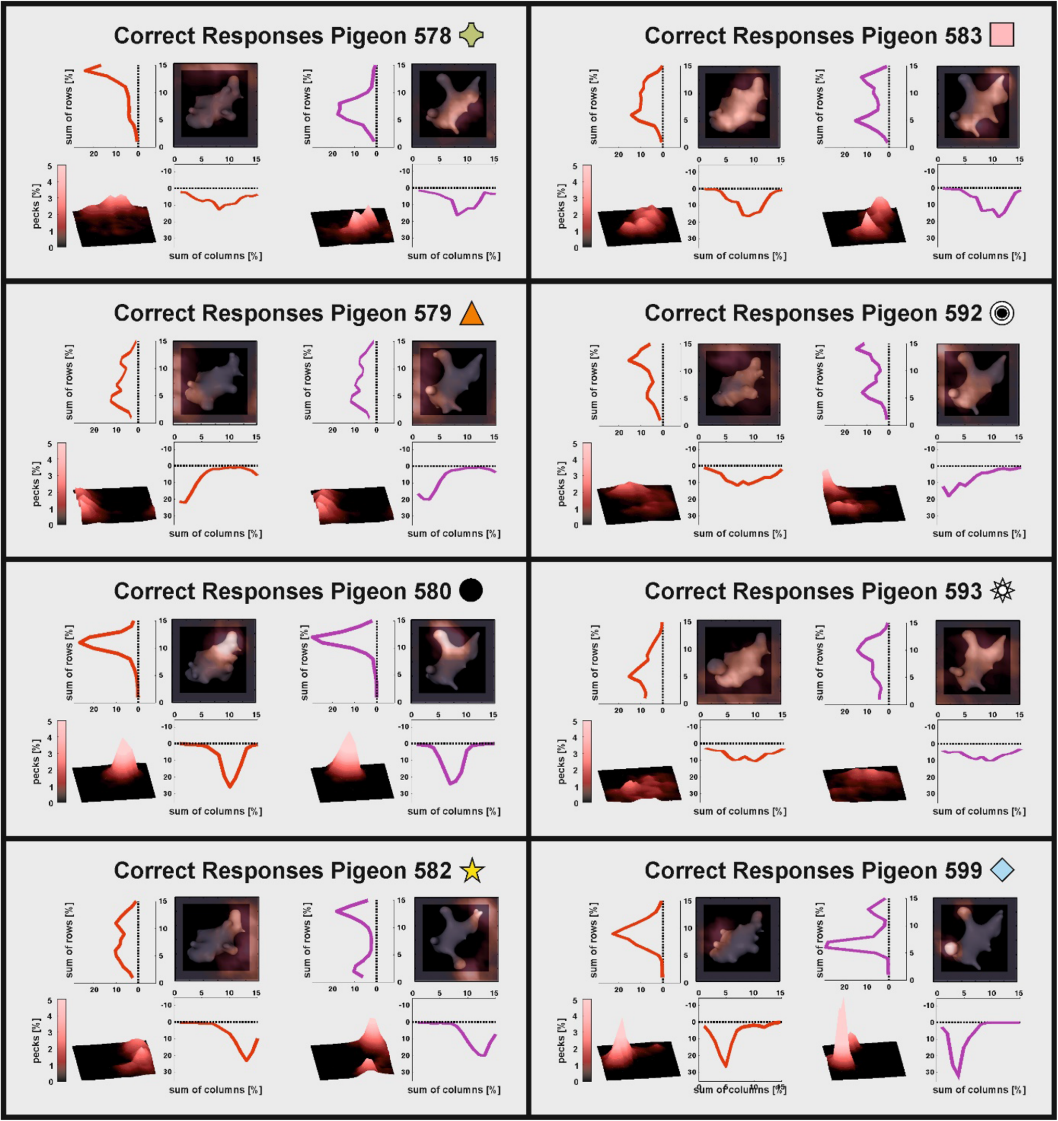
Heatmap analysis for all individual pigeons. Only pecks from correct trials for the two stimulus classes (left: X, right: Y) are shown. Additional analyses as presented in Fig. 3 are given in the supplementary figures

Animals preferred different embryo features indicated by their idiosyncratic pecking locations for the two stimulus classes (Fig. 6).

Since individual pecks often came from the same trial, we validated our analysis only using single pecks from a particular trial to rule out that our results were influenced by possible dependencies of pecks within a trial. To this end, we only chose one random peck event from each trial as a valid event from which we randomly drew 250 training and 250 test pecks for classification. Results were virtually indistinguishable from our previous approach using all pecking events indicating that this approach was valid and not influenced by peck dependencies within the trial (P578: 81.28%, P579: 51.88%, P580: 80.92%, P582: 69.96%, P583: 63.84%, P592: 76.48%, P593: 67.12%, P599: 79.68%, see supplementary Fig. 12). For that reason, all following analyses were computed including all pecks from all trials to increase the number of available data points.

**Fig. 7:**
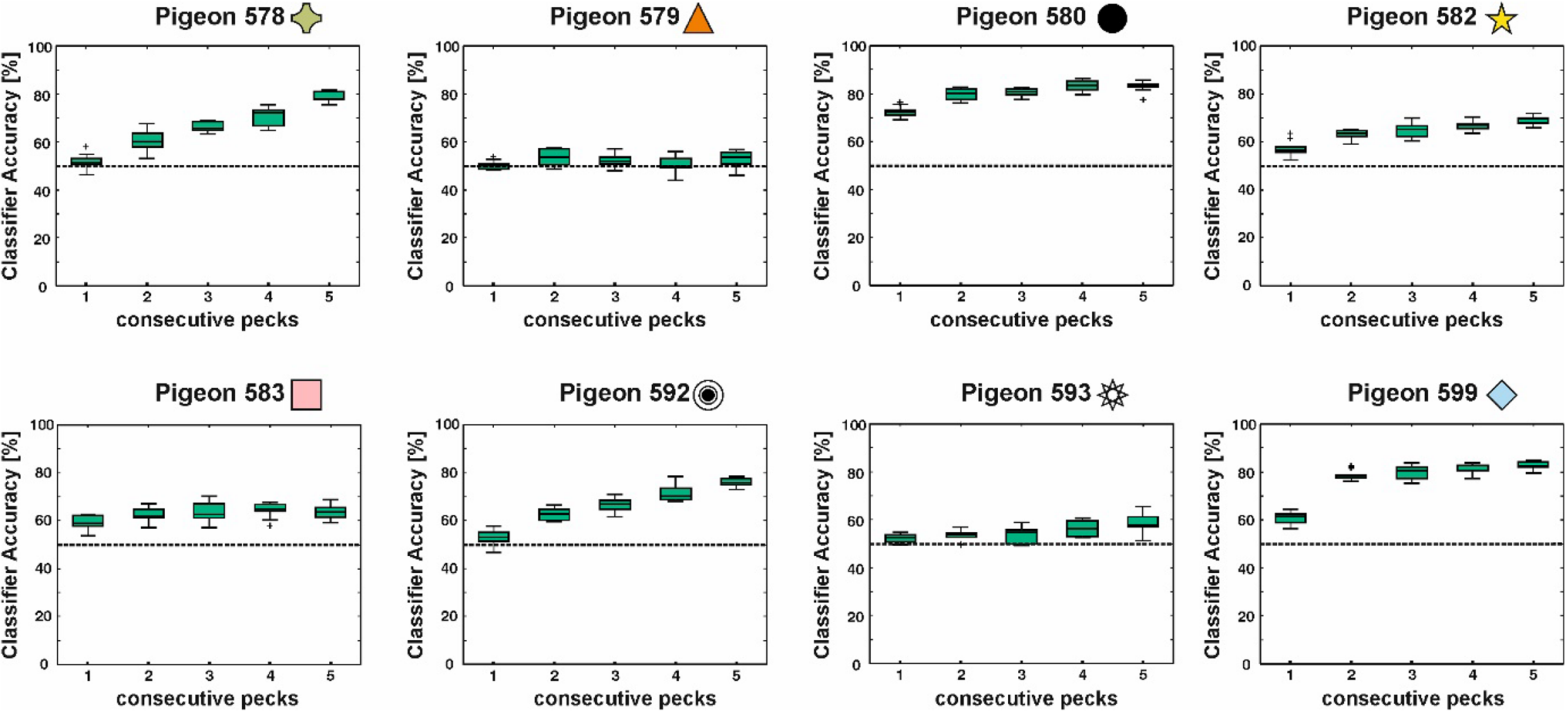
Single peck analysis. The classifier response for the known stimuli is depicted for each animal and for each of the five consecutive pecks. For the majority of the animals, the classification accuracy increases for consecutive pecks. Boxplots represent the lower quartile (Q1), the median and the upper quartile (Q3). Whiskers represent Q1 − 1.5 * IQR and Q3 + 1.5 * IQR

In a secondary analysis, we were also interested whether the first peck was already sufficient for stimulus classification as has been demonstrated by Cook et al. (2005) in a spatial choice task. Interestingly, using only the first peck for classification resulted in overall worse classification accuracy compared to the following pecks (see Fig. 7). For almost all pigeons, classification accuracy increased with consecutive pecks on the sample stimulus indicating that features of interest were not immediately pecked on after stimulus onset.

For transfer-stimuli trials (Fig. 5), we found identical results compared to known-stimuli trials (P578: 82.09%, P579 = 53.48%, P580: 83.68%, P582: 65.52%, P583: 64.96%, P592: 76.12%, P593: 63.16%, P599: 76.20%, all *p*s < 0.001). There was no difference in classification accuracy from known-stimuli trials in CC accuracy for any pigeon.

**Fig. 8:**
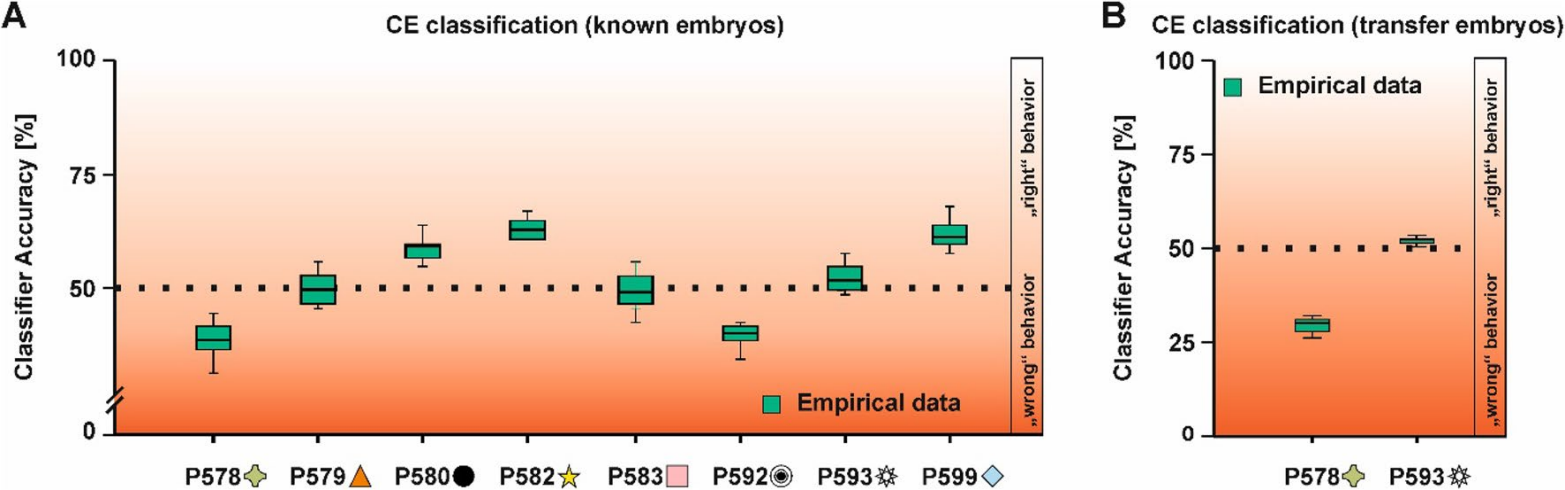
CE (correct–error) classification results of the second experimental stage. **A** shows CE classification for the known stimuli. **B** depicts the CE classification for the transfer stimuli. Dark red colors indicate that the pigeons showed the “wrong” behavior for the respective stimulus class and thus a confusion between the categories X and Y. Light red colors indicate that the animals showed the “right” behavior for the respective stimulus class resulting in a confusion within a given category in the CE classification. Boxplots represent the lower quartile (Q1), the median and the upper quartile (Q3). Whiskers represent Q1 − 1.5 * IQR and Q3 + 1.5 * IQR (color figure online)

For all eight animals, there were sufficient errors allowing for a correct–error or CE classification in known-stimuli trials in which the classifier was trained with correct trials and tested on error trials. Here, two pigeons demonstrated similar pecking patterns in correct and error trials as the classifier yielded above chance results in a CE classification (P582: 62.36%, P599: 61.16%, all *p*s < 0.001, Fig. 8A, for the classification accuracy based on shuffled input data see supplementary Fig. 13). Pecking in error trials was more dispersed compared to correct trials resulting in a reduced accuracy for CE compared to CC classification for both pigeons (all *p*s < 0.001). This finding was, however, not exclusive for animals that pecked on similar locations during correct and error trials. Seven out of eight pigeons demonstrated significant differences when comparing the cumulative distributions of pecking (pooled across class X and Y) across the stimulus squares between correct and error trials (578: *D* = 0.12, *p* = 0.003, 579: *D* = 0.19, *p* < 0.001, 580: *D* = 0.47, *p* < 0.001, 582: *D* = 0.35, *p* < 0.001, 583: *D* = 0.35, *p* < 0.001, 592: *D* = 0.19, *p* < 0.001, 599: *D* = 0.49, *p* < 0.001, see supplementary Fig. 14). Results for class X and Y individually can be found in supplementary Table 2.

For pigeons P578 and P592, we found that CE classification was significantly below chance (P578: 38.12%, P592: 38.96%, both *p*s < 0.001). This type of pecking behavior indicates that the pigeons showed the “wrong” behavior for the respective stimulus class (e.g. they pecked on class Y features during class X trials and vice versa). Thus, they recognized the stimulus classes, but mixed up their identity in error trials.

The last three pigeons demonstrated no significant difference from chance in the CE classification (P579: 49.16%, P583: 49.00%, P593: 52.24%, all *p*s > 0.174) indicating that there was no relationship between correct and error trial pecking locations. For two pigeons, sufficient errors were made in transfer-stimuli trials enabling further analysis. For P578, we again found a significantly lower classification accuracy compared to chance [29.64%, *t*(9) = 12.64, *p* < 0.001, Cohen’s *d* = 3.97, Fig. 8B]. For pigeon P593, there was no association between correct and error trial pecking locations [52.70%, *t*(9) = 1.55, *p* = 0.156, Cohen’s *d* = 0.49]. Both pigeons, thus, showed consistent patterns in known-stimuli and transfer-stimuli trials.

Error–error or EE classification in which the classifier was both trained and tested on pecking events during erroneous trials could be performed for all animals in known-stimuli trials. Aim of the analysis was to quantify the internal consistency in pecking during error trials. It was above chance for five of the eight animals (P580: 63.16%, P582: 61.64%, P583: 74.44%, P592: 57.52%, P599: 72.96%, all *p*s < 0.004) indicating that pecking locations in error trials were to a certain degree consistent rather than random. A consistent response pattern in error trials was predominantly found in birds in which the C–E classification also yielded above or below chance level results, validating our interpretation of the pecking behavior during error trials. For pigeons P578, 579 and P593, no significant difference from chance could be found (all *p*s > 0.025). For pigeons P578 and P593, there were sufficient error events in transfer-stimuli trials to allow for EE classification. Interestingly, classification accuracy was above chance for pigeon P578 [67.6%, *t*(9) = 5.95, *p* < 0.001] in known-stimuli trials. For pigeon 593, it remained at chance level [53.92%, *t*(9) = 1.29, *p* = 0.228].

## Discussion

In the present study, we investigated perceptual categorization learning in pigeons using “digital embryos” as visual stimuli. The process of “virtual phylogenesis” can generate any number of these artificial stimuli and categories while maintaining natural stimulus characteristics. We hypothesized that pigeons can both (1) learn to categorize digital embryos of different stimulus classes as well as (2) transfer their knowledge onto novel exemplars of these classes. Our results clearly demonstrate that all animals were able to discriminate between classes X and classes Y and could transfer this knowledge to never before encountered stimuli of the same class. Finally, we hypothesized that (3) pecking behavior is indicative of the pigeon’s attention towards critical stimulus features and that we could therefore predict the presented stimulus class solely based on pecking locations. Indeed, we could decode the presented stimulus class for the majority of the pigeons (7 out of 8) using a k-nearest neighbor approach. Classification accuracy was highly comparable between training and transfer trials. However, classification accuracy was not uniform across animals with some pigeons showcasing high accuracy classification results whereas other pigeons showed lower classification accuracy that was nonetheless significantly different from chance level.

Peck tracking using touch-screen technology serves similar purposes as eye tracking in primate studies. Pigeons track relevant aspects of complex visual displays in discrimination tasks by pecking on them (Castro and Wasserman 2014, 2017; Dittrich et al. 2010). Thus, the systematic analysis of the pecking behavior can give insight into attentional mechanisms. On the descriptive level, we found that most animals favored different features of the digital embryos as there was little overlap among animals regarding the most pecked area for stimulus classes if pecking is used as a proxy for attention (Fig. 6). This strongly suggests that pigeons rely on local stimulus features, in line with the well documented “local precedence effect” in pigeons (Cavoto and Cook 2001; Cerella 1980). In addition, Yamazaki et al. (2007) found that pigeons can categorize highly scrambled pictures of humans based on small local features, thereby neglecting the overall stimulus configuration. In a comparative approach, Aust and Braunöder (2015) conducted both an exemplar and rule-based categorization task in pigeons and humans and found that pigeons rely on local features whereas humans have no strict preference for local or global information to solve the tasks. However, a variety of studies also found global strategy-based categorization in pigeons, indicating that they are capable of using both strategies (e.g., Goto et al. 2004; Yamazaki et al. 2007). Possible explanations for this discrepancy might be due to differences in experimental procedures and related task-demands. Goto et al. (2004) found that pigeons prefer global strategies if local information is densely packed in the stimulus material. Dense packing of elements has been demonstrated to promote global preference effects in humans (Dukette and Stiles 2001). Since our stimuli did not feature dense stimulus elements, a local approach was likely to be expected. However, due to the small size of our stimulus display, the resulting peck distribution might obscure the exact controlling stimulus features and thus not represent a direct reflection of the focus of the pigeons’ attention. Further studies using larger stimulus displays are needed to directly pinpoint the controlling stimulus features the animals use.

While a descriptive analysis of pecking location can provide insight into the use of local or global preferences, it cannot illuminate on the underlying learning strategy to solve the task. Our classifier analysis might indicate different strategies of the animals with regards to how many categories were likely learned during the categorization process. Since our task employed a force choice procedure, the categorization could theoretically be solved by only learning about one stimulus class. For example, if only class X had been learned, the animal simply needed to identify if the stimulus is of class X or not X to determine the upcoming choice. In the CC classification, such a learning pattern might correspond to an intermediate classification accuracy (roughly between 60 and 70%) as the animals demonstrated a clear pecking pattern for one stimulus class in correct trials, but a rather unfocused pecking pattern for the other stimulus class. If both stimulus classes received specific pecks onto relevant class features, classification accuracy should jump to levels around 80% since the classifier was trained with information about class X and Y rather than a single class. The classifier performance for four of our pigeons (578, 580, 592, 599) ranged around 80% and thus, they might have learned about both stimulus classes. This might also be reflected in the above chance classification accuracy in the EE classification in most of these birds. Three pigeons (582, 583, 593) might have payed attention to features of one stimulus class as the classification accuracy ranged between 60 and 70%. However, to ultimately decide between these different strategies, additional experiments featuring larger stimuli are required. Interestingly, one pigeon pecked on the left side of all stimuli, regardless of the presented stimulus class (see supplementary Fig. 4), but still performed the task at a very high level. Pigeons are known to have a superiority for object discrimination using their right eye/left hemisphere and it is conceivable that this contributed to this lateralized behavior (Güntürkün 1985; Yamazaki et al. 2007). Most importantly, this observation shows that there is no absolute necessity to peck onto informative features to eventually perceive and process them. Since this was, however, only one animal, it seems to be the exception rather than the rule.

We furthermore used the classifier to inform about why errors occur in specific animals by training it with correct trials and testing it on error trials (CE classification). We found that for three animals (580, 582, 599) during the transfer test phase, CE classification simply dropped the classification accuracy, but it remained significantly above chance (~ 60%). These animals showed the “right” behavior for the respective stimulus class (e.g. they pecked on class X features during class X trials) and pecked onto similar features in error trials as in correct trials. However, pecking on these features was less focused (see supplementary Fig. 14) probably indicating a lack of attention (Dittrich et al. 2010). It has been shown in behavioral experiments that the choice performance of pigeons increases with prolonged stimulus presentation times. These results in conjunction with behavioral and electrophysiological properties of neurons in the nidopallium caudolaterale suggest that pigeons integrate sensory information over time (Lengersdorf et al. 2014; Wittek et al. 2021). Thus, the animals might have failed accumulating sufficient evidence to determine the correct class membership to terminate in a correct response instead leading to a failure in categorization. Two animals demonstrated inversed pecking patterns whenever they made an error as indicated by a below chance classification (~ 35%). Thus, they showed the “wrong” behavior for the respective stimulus class (e.g. they pecked on class Y features during class X trials and vice versa). In this specific case, the animals mix-up the two classes. Obviously, these kind of errors occurred only in animals that learned about both categories according to the CC classification. Finally, three animals showed no difference from chance-level performance in CE classification indicating that they pecked randomly during error trials possibly reflecting the failure to recognize any class-specific features.

Our results fit well into the contemporary view of categorization learning in pigeons (Güntürkün et al. 2018; Soto and Wasserman 2010). Pigeons used several, different stimulus features to solve the categorization task. Individual differences in the selection of local stimulus features, as present in our pigeons, are in line with previous reports that used peck tracking to indicate the areas of attention in complex stimulus displays. Jitsumori and Yoshihara (1997) reported different strategies in individual pigeons when categorizing angry and happy facial expressions. Some pigeons attended more to the area of the eyes, some more to the mouth.

Peck location data indicating that individual pigeons focusing on different parts of the stimulus (Fig. 6; e.g. P582, P592, P599) tell us that multiple features are evaluated within a single subject. However, the features that individual pigeons pecked on were subject-specific. These category specific stimulus features might be discernable from neuronal population responses in visual associative areas in the pigeon brain (Azizi et al. 2019; Koenen et al. 2016). In addition, Castro and Wasserman (2017) found in their experiments that with increasing choice accuracy, pecks directed onto relevant features of the categories increased. Thus, during learning the amount of attention to reward predicting cues increased and was clearly signaled by the pecking behavior of the animals. Based on reward contingencies associated with the resulting responses, the feature that was the best predictor of reward becomes the feature that predominantly, but not exclusively controlled the pigeon’s behavior (Castro et al. 2020). This kind of feature selection does not seem to be based on feature configuration but rather on the additive integration of individual features or common elements (Jitsumori and Yoshihara 1997; Soto and Wasserman 2010). This process strictly follows predictions of influential learning theories (e.g., Rescorla and Wagner 1972) and can be explained by a dopamine-mediated reduction of associated prediction errors (Schultz 1998) that have also been demonstrated analogously in the pigeon brain (Packheiser et al. 2021). Thus, the consistent behavioral performance of individual pigeons in categorizing digital embryos might be based on common stimulus elements and their reward prediction acquired during perceptual categorization learning.

Taken together, our study provides insights about both the center of attention in pigeons during categorization as well as the underlying learning strategy using a machine learning approach. Pigeons successfully transferred class knowledge to members of each embryo “family” or class that they hav not seen before, using multiple strategies to do so. Generally, most animals used a local approach with the exception of one animal that did not peck on relevant features at all. Interestingly, there has been a strong record of asymmetrical lateralization in birds proclaiming that the left hemisphere is dominant in local feature extraction whereas the right hemisphere is dominant in processing global feature extraction (Yamazaki et al. 2007; Güntürkün et al. 2020). It would be interesting to investigate in future studies if the behavioral strategy could be altered by simple eye-occlusion procedures to bias the processing of a particular hemisphere in birds. Regarding the learning strategy, a number of animals used the dichotomous nature of the task by likely only learning about one category. It would be highly interesting to investigate the corresponding neural correlates of these different strategies and to see how relevant visual associative layers such as the NFL (Koenen et al. 2016) and MVL (Azizi et al. 2019) encode stimulus classes with respect to the chosen categorization strategy.

## Supplementary Information

Below is the link to the electronic supplementary material.

In total, four pigeons conducted the first stage of the transfer test phase in which the transfer-stimuli trials did not yield any feedback such as reward or punishment. Performance in known-stimuli and transfer-stimuli was generally high (Mean = 93.49 %, SD = 4.35 %; Mean = 92.31 %, SD = 3.31 %, respectively, see supplementary figure 15) and was significantly different from chance (t(3) = 2256.53, *p* < .001, Cohen’s d = 10.00; t(3) = 2964.65, *p* < .001, Cohen’s d = 12.78, respectively). There was no difference in performance between known-stimuli and transfer-stimuli trials (t(3) = 0.83, *p* = > .250, Cohen’s d = 0.35). For the stimulus classes individually, both class X (known-stimuli trials mean = 94.08 %, SD = 5.36 %; transfer-stimuli trials mean = 91.47 %, SD = 6.07 %) and class Y (known-stimuli trials mean = 92.89 %, SD = 3.70 %; transfer-stimuli trials mean = 93.10 %, SD = 1.81 %) showed above chance-level performance (all *p*s < .001) with no difference between known- and transfer-stimuli trials (all *p*s > .250). There was no difference between stimuli of class X and Y in both known- (t(3) = 0.79, *p* > .250, Cohen’s d = 0.37) and transfer-stimuli trials (t(3) = 0.53, *p* = > .250, Cohen’s d = 0.33). For the four animals that were tested in non-reinforced transfer conditions, we found that the CC classifier could predict the presented stimulus class above chance-level in known-stimuli trials (P580: 84.16 %, P592: 74.16 %, P593: 66.80 %, P599: 78.20 %, all *p*s < .001, see supplementary figure 16A). CC classification accuracy was also above chance-level in transfer-stimuli trials (P580: 84.48 %, P592: 71.28 %, P593: 61.60 %, P599: 76.68 %, all *p*s < .001, see supplementary figure 16B). Classification accuracy did not differ in transfer- compared to known-stimuli trials except for pigeon P593. Here, classification accuracy dropped significantly in transfer-stimuli trials (t(9) = 3.77, *p* = .004, Cohen’s d = 1.20). For pigeons P592 and P593, we could also analyze known-stimuli error trials as there were sufficient error events to warrant further analysis. For pigeon P592, we found a significantly lower than chance classification accuracy when using pecks from correct trials as class predictors and error pecks as test events (35.24 %, t(9) = 9.33, *p* < .001, Cohen’s d = 2.96, see supplementary figure 16C). This indicated that P592 pecked on features relevant for stimulus class X even though a class Y stimulus was presented in error trials and vice versa (see supplementary data for the pecking responses of individual pigeons). There was no difference from chance in the CE classification (t(9) = 1.76, *p* = .112, Cohen’s d = 0.55) for pigeon P593 indicating that there was no association between pecking locations of correct and error trials. For neither pigeon, transfer-stimuli trials could be analyzed in the CE classification due to low number of error trials. There were sufficient errors in known-stimuli trials for pigeons P592 and P593 to perform an EE classification in which the classifier was both trained and tested on pecking events during erroneous trials. For both pigeons, we found no evidence that pecking in error trials could be predict the presented stimulus class in other error trials (P592: 56.56 %, P593: 52.92 %, both ps >. 085). There were not sufficient error events in transfer-stimuli trials preventing further analysis. (DOCX 5350 KB)
