# Supplementary material for: Digital embryos: a novel technical approach to investigate perceptual categorization in pigeons (Columba livia) using machine learning
Source: Anim Cogn. 2022 Jan 6;25(4):793–805. doi: 10.1007/s10071-021-01594-1 (PMC9334434; doi:10.1007/s10071-021-01594-1)
Supplement: Supplementary file 1 — In total, four pigeons conducted the first stage of the transfer test phase in which the transfer-stimuli trials did not yield any feedback such as reward or punishment. Performance in known-stimuli and transfer-stimuli was generally high (Mean = 93.49 %, SD = 4.35 %; Mean = 92.31 %, SD = 3.31 %, respectively, see supplementary figure 15) and was significantly different from chance (t(3) = 2256.53, p < .001, Cohen’s d = 10.00; t(3) = 2964.65, p < .001, Cohen’s d = 12.78, respectively). There was no difference in performance between known-stimuli and transfer-stimuli trials (t(3) = 0.83, p = > .250, Cohen’s d = 0.35). For the stimulus classes individually, both class X (known-stimuli trials mean = 94.08 %, SD = 5.36 %; transfer-stimuli trials mean = 91.47 %, SD = 6.07 %) and class Y (known-stimuli trials mean = 92.89 %, SD = 3.70 %; transfer-stimuli trials mean = 93.10 %, SD = 1.81 %) showed above chance-level performance (all ps < .001) with no difference between known- and transfer-stimuli trials (all ps > .250). There was no difference between stimuli of class X and Y in both known- (t(3) = 0.79, p > .250, Cohen’s d = 0.37) and transfer-stimuli trials (t(3) = 0.53, p = > .250, Cohen’s d = 0.33). For the four animals that were tested in non-reinforced transfer conditions, we found that the CC classifier could predict the presented stimulus class above chance-level in known-stimuli trials (P580: 84.16 %, P592: 74.16 %, P593: 66.80 %, P599: 78.20 %, all ps < .001, see supplementary figure 16A). CC classification accuracy was also above chance-level in transfer-stimuli trials (P580: 84.48 %, P592: 71.28 %, P593: 61.60 %, P599: 76.68 %, all ps < .001, see supplementary figure 16B). Classification accuracy did not differ in transfer- compared to known-stimuli trials except for pigeon P593. Here, classification accuracy dropped significantly in transfer-stimuli trials (t(9) = 3.77, p = .004, Cohen’s d = 1.20). For pigeons P592 and P593, we could also analyze known-stimul [file 10071_2021_1594_MOESM1_ESM.docx]

**Supplementary figures**


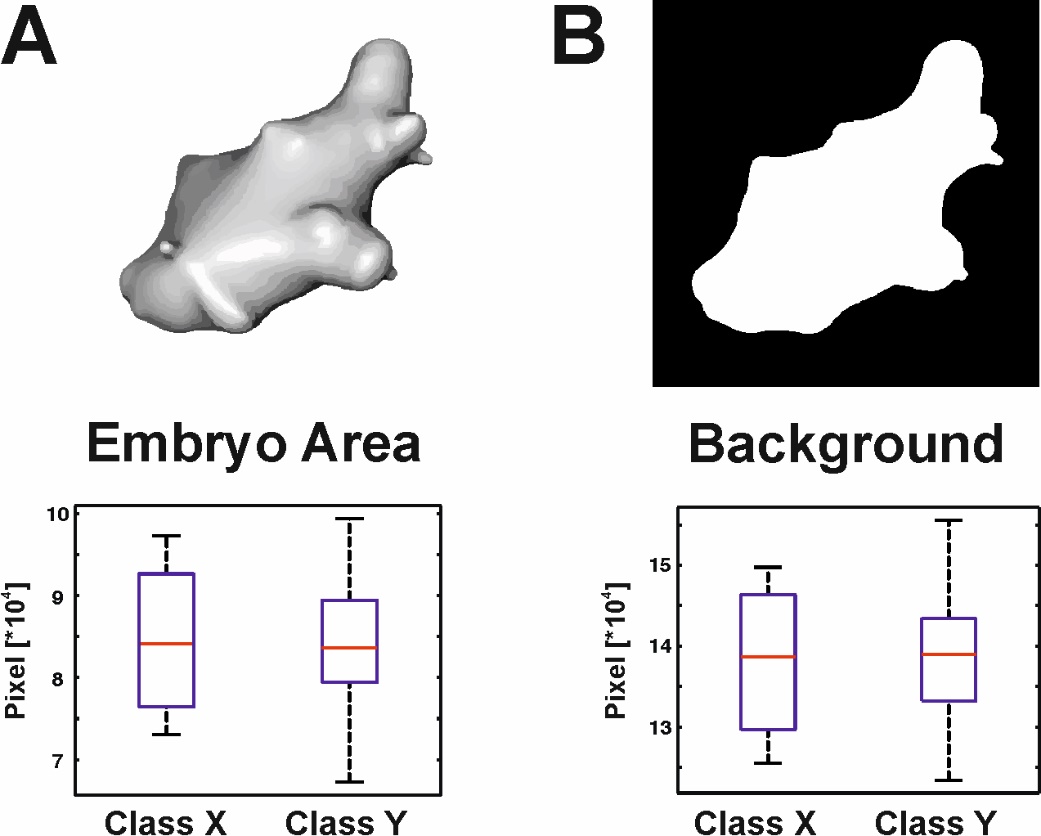


**Supplementary figure 1. Measurement of the stimulus surface area and the surrounding background. A.** Distribution of the surface area of single stimuli of each class (area measured in pixel). **B.** Distribution of the background surface of single stimuli of each class (area measured in pixel). Boxplots represent the lower quartile (Q1), the median and the upper quartile (Q3). Whiskers represent Q1 - 1.5 * interquartile range (IQR) and Q3 + 1.5 * IQR.


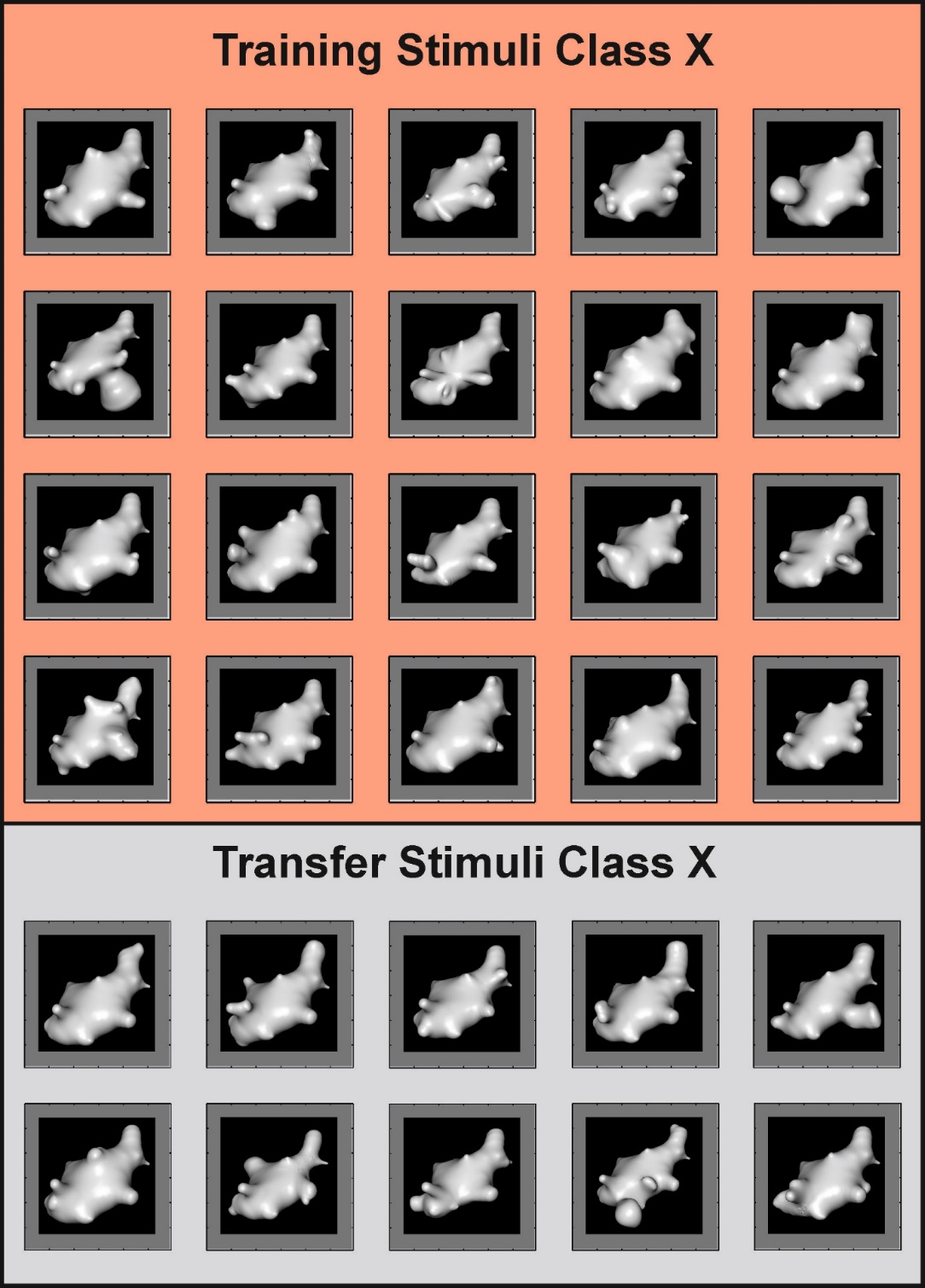


**Supplementary figure 2. Example training and transfer stimuli for class X from one session.**


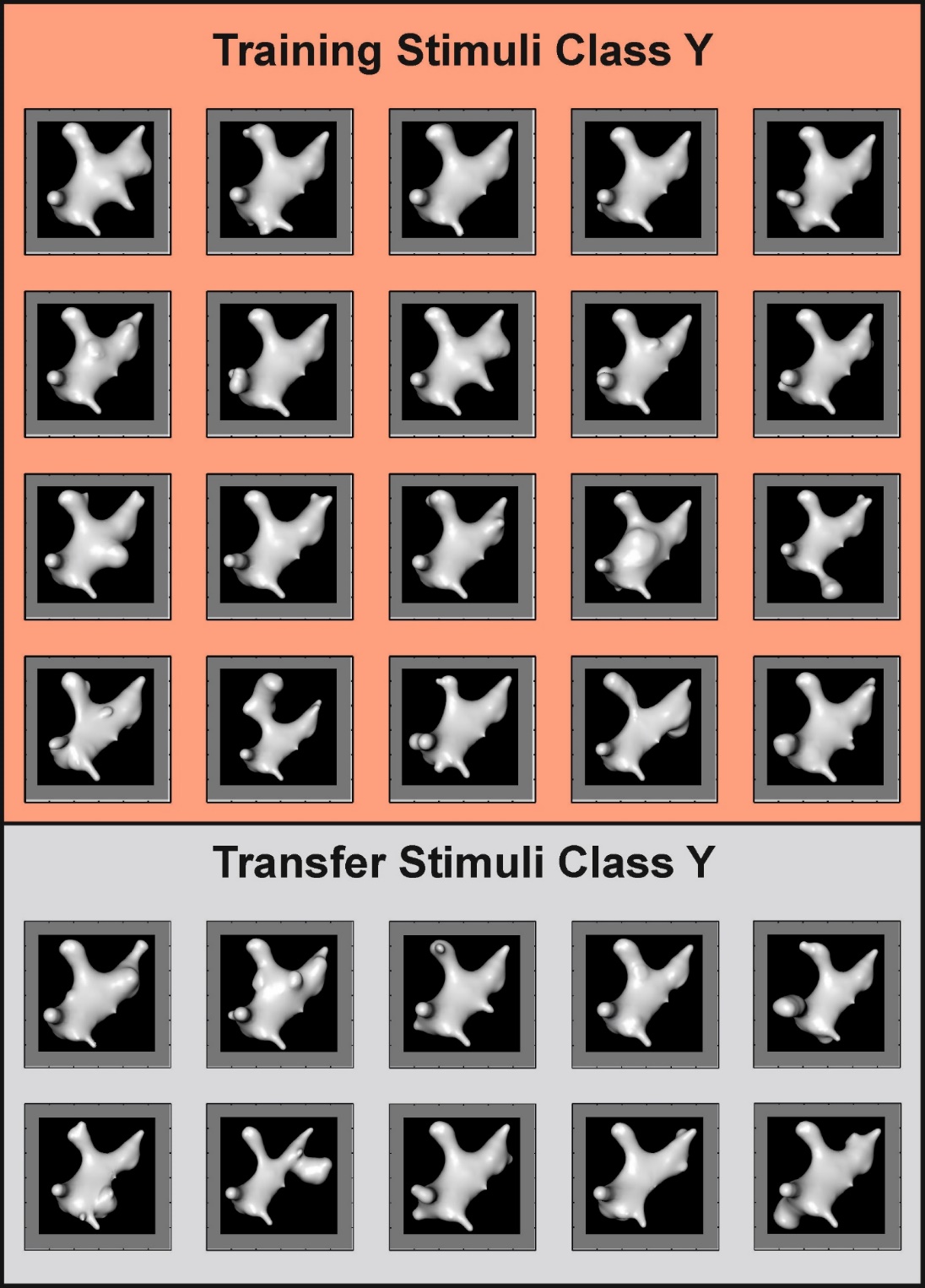


**Supplementary figure 3. Example training and transfer stimuli for class Y from one session.**


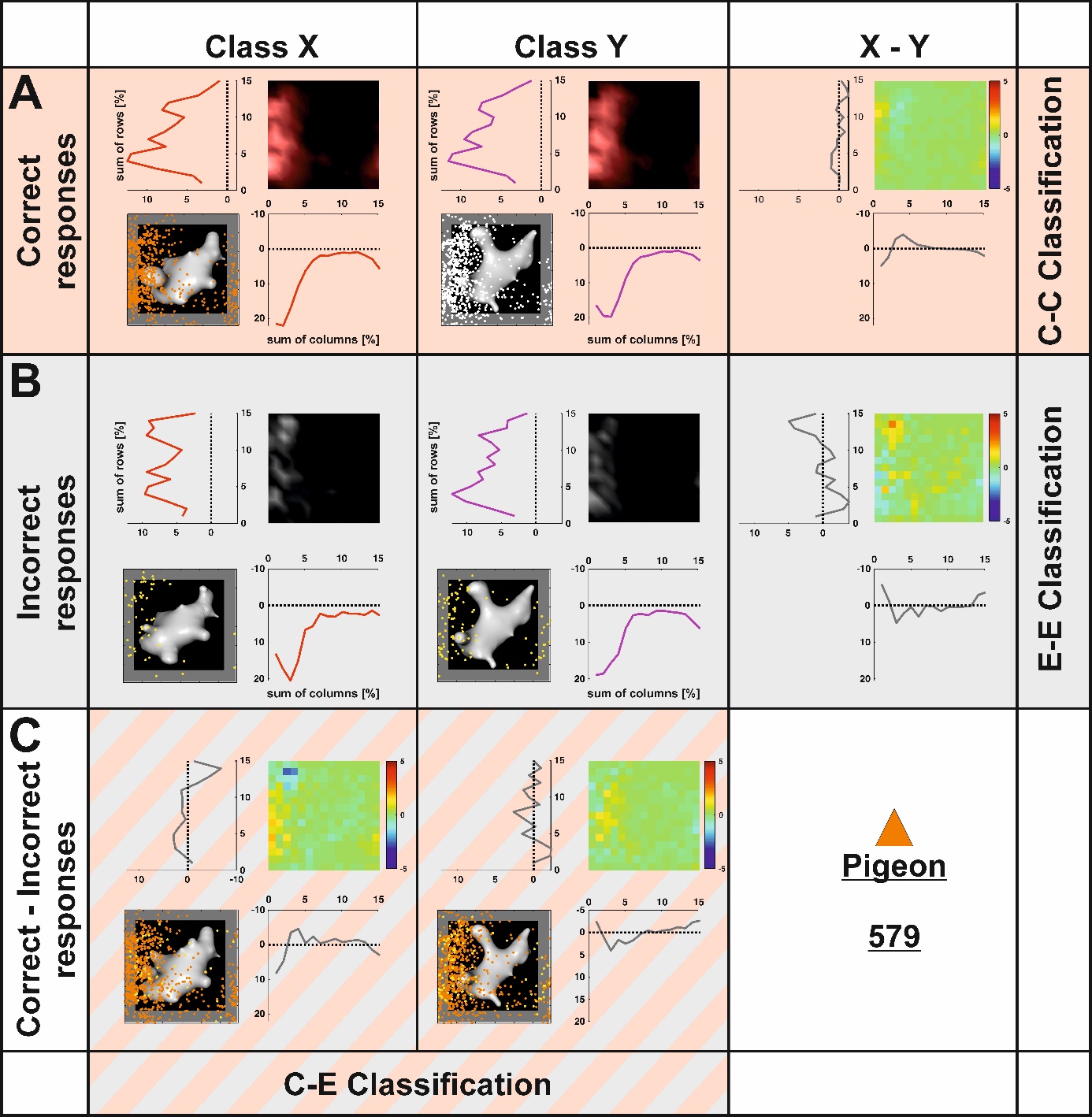


**Supplementary figure 4. Heatmap analysis for pigeon 579.**


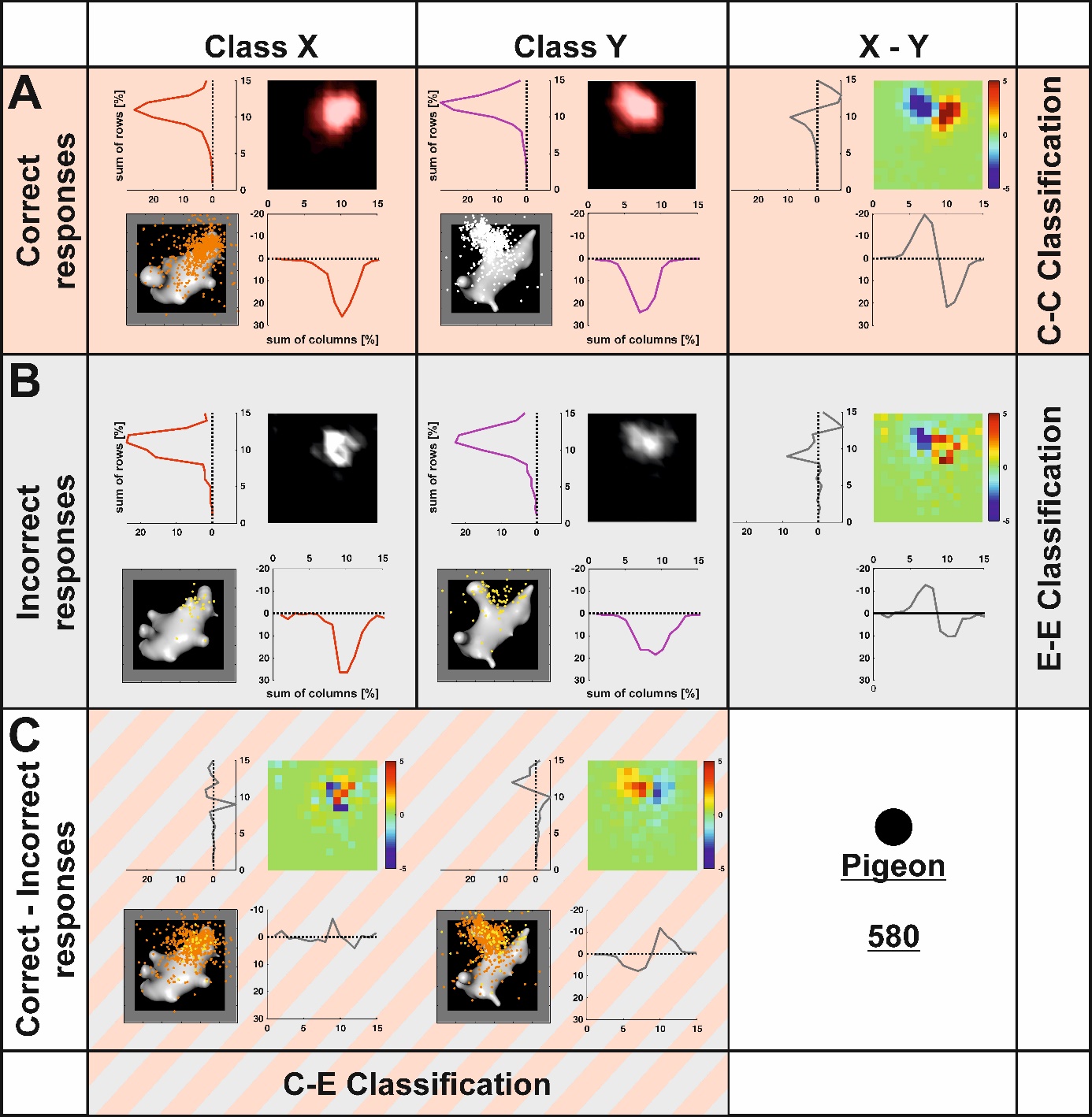


**Supplementary figure 5. Heatmap analysis for pigeon 580.**


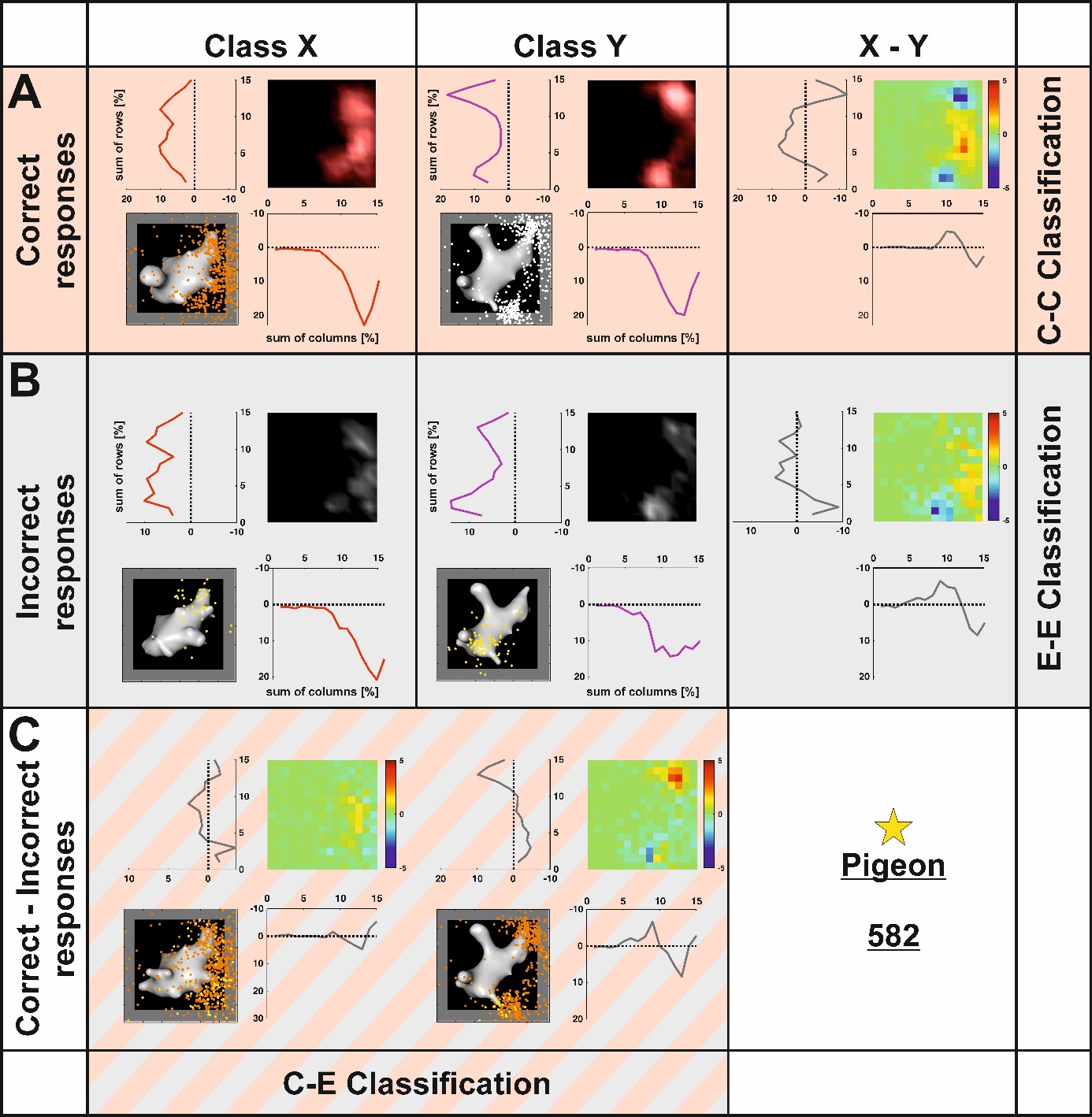


**Supplementary figure 6. Heatmap analysis for pigeon 582.**


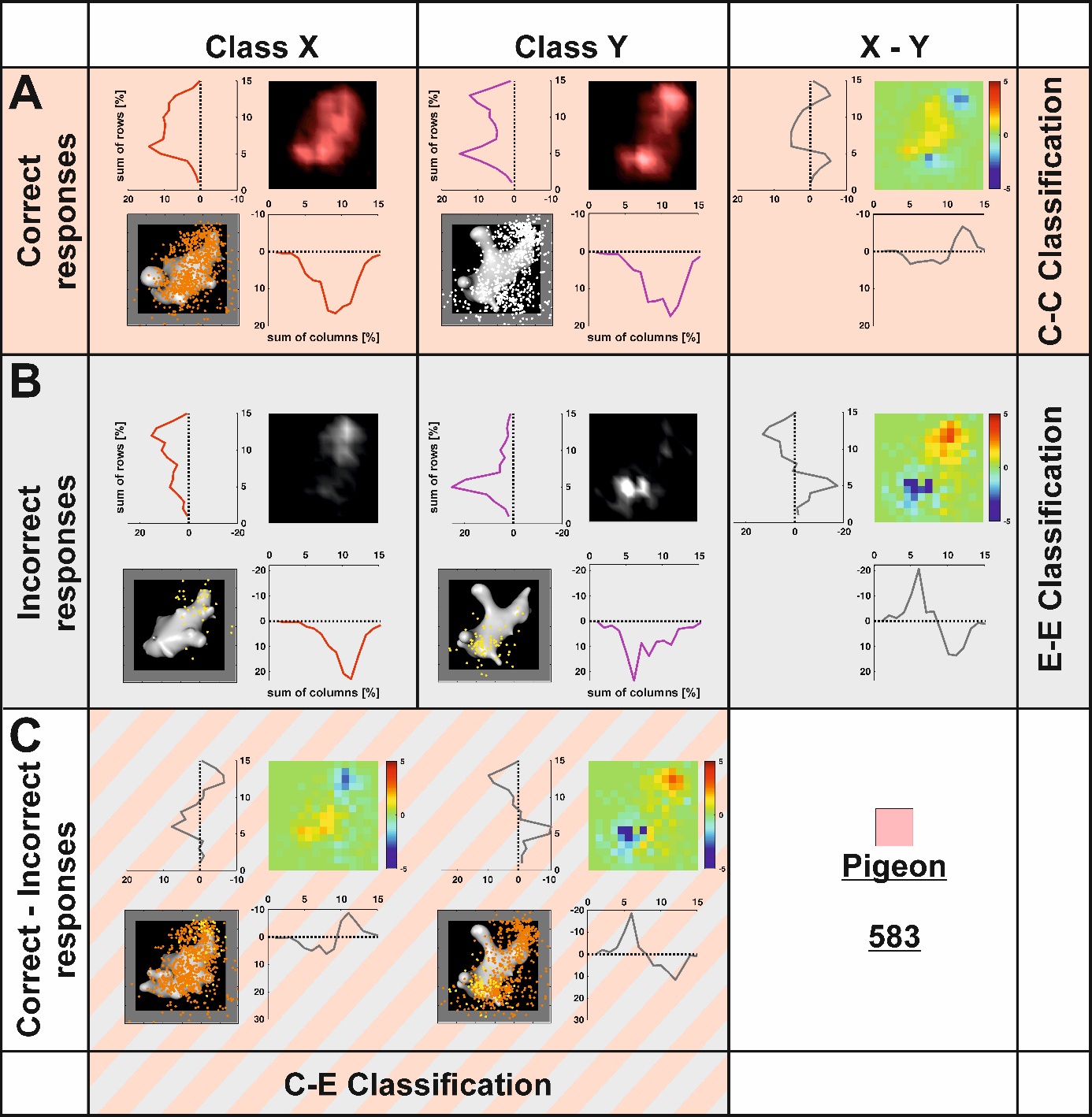


**Supplementary figure 7. Heatmap analysis for pigeon 583.**


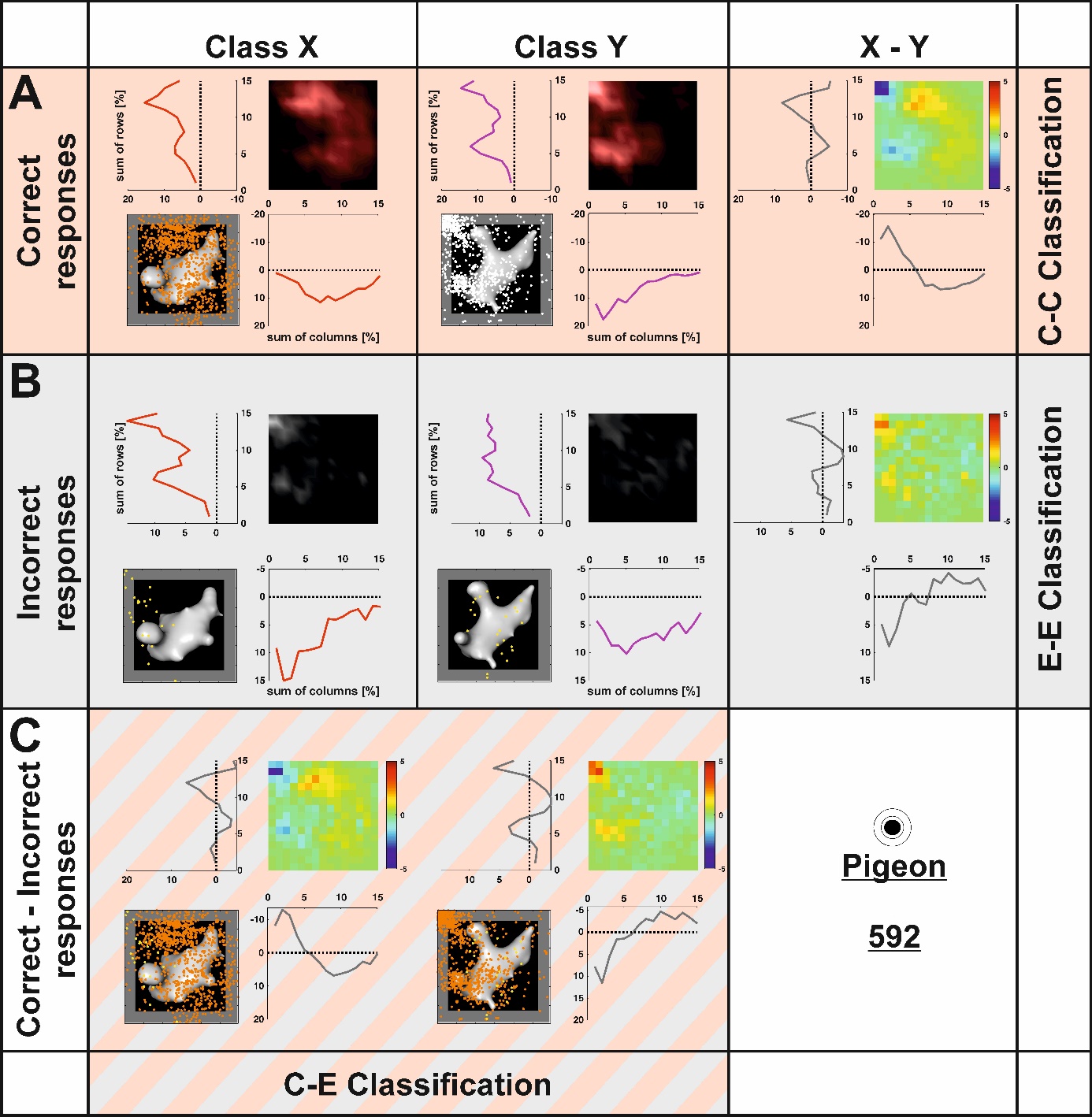


**Supplementary figure 8. Heatmap analysis for pigeon 592.**


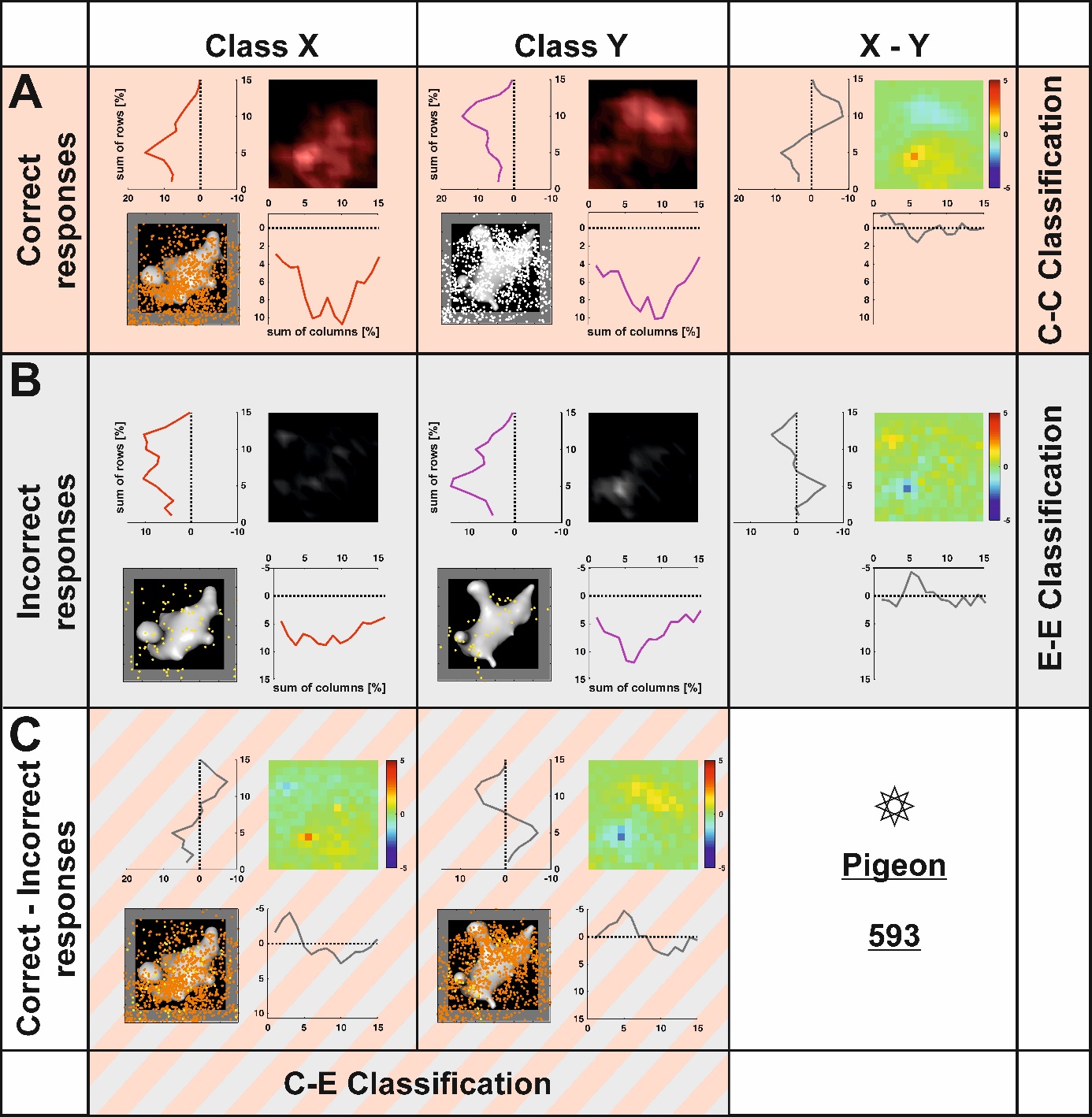


**Supplementary figure 9. Heatmap analysis for pigeon 593.**


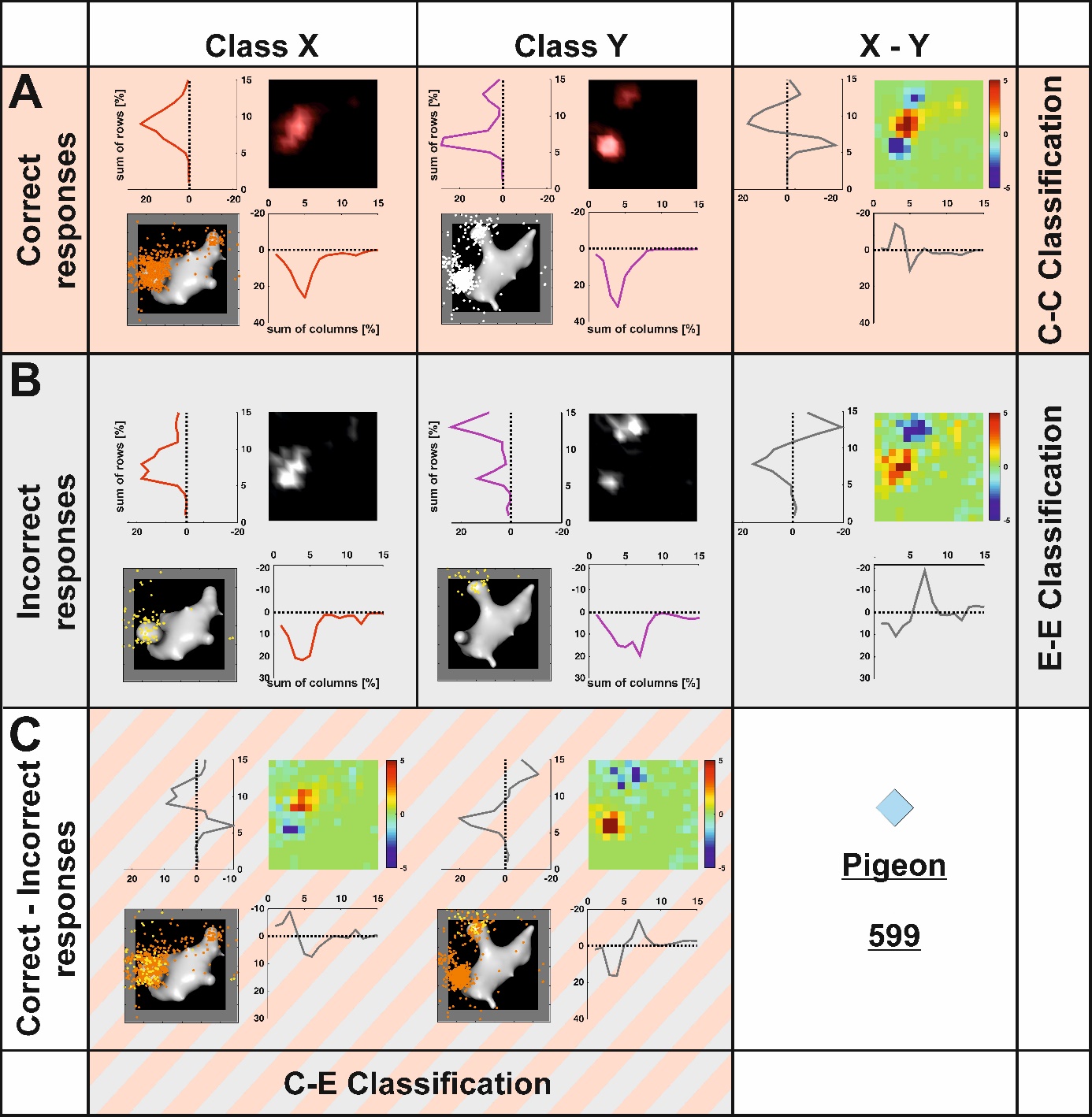


**Supplementary figure 10. Heatmap analysis for pigeon 599.**

**
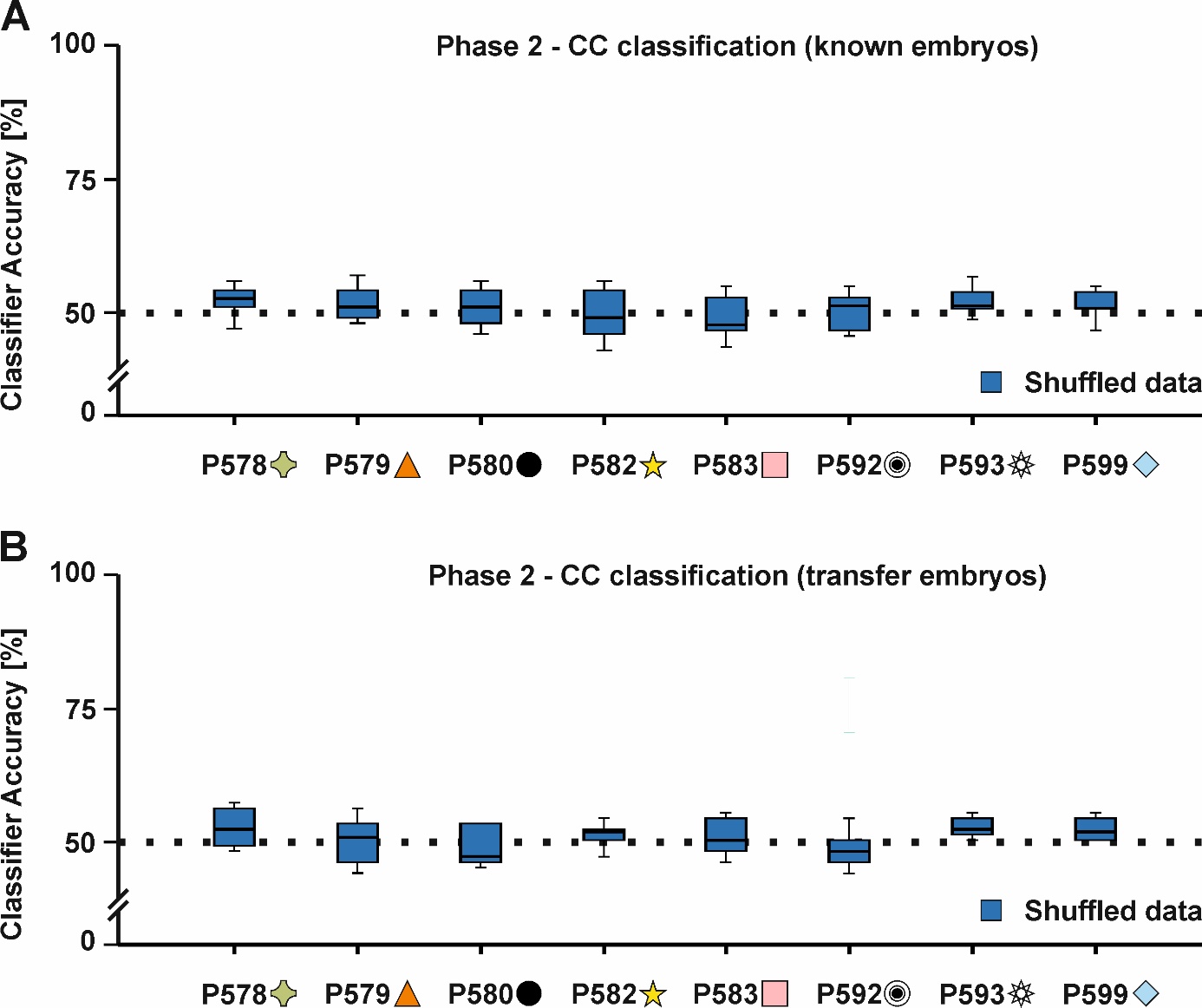
**

**Supplementary figure 11. CC classification results of the second experimental stage for shuffled data.** Boxplots represent the lower quartile (Q1), the median and the upper quartile (Q3). Whiskers represent Q1 - 1.5 * IQR and Q3 + 1.5 * IQR.


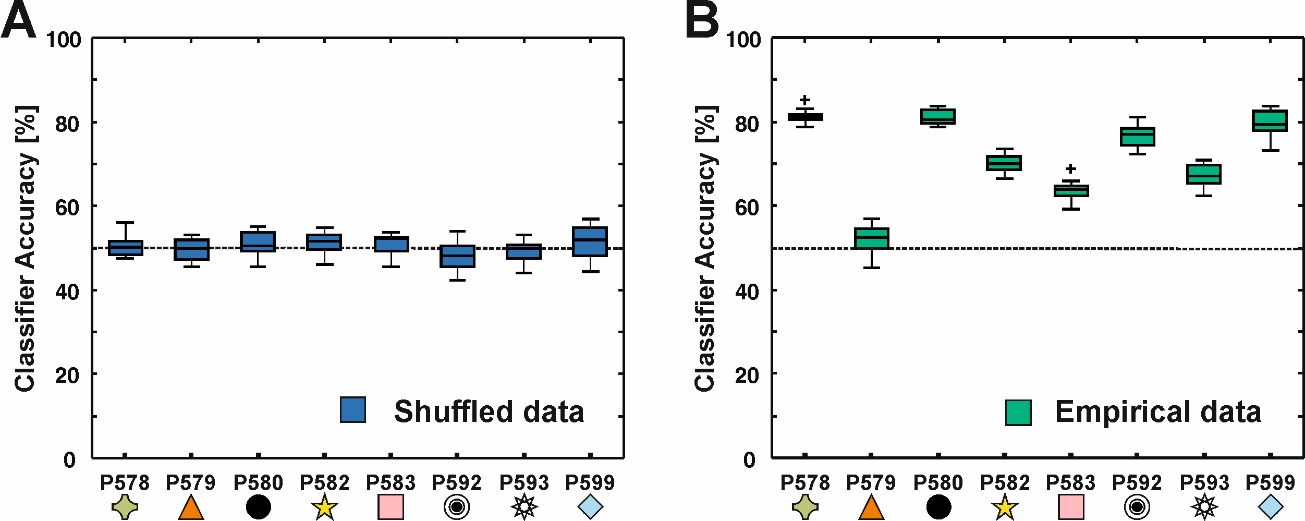


**Supplementary figure 12. CC classification results using only a single peck of each trial**

**A** shows the classifier response for shuffled data **B** depicts the classifier results for empirical results. Boxplots represent the lower quartile (Q1), the median and the upper quartile (Q3). Whiskers represent Q1 - 1.5 * IQR and Q3 + 1.5 * IQR.


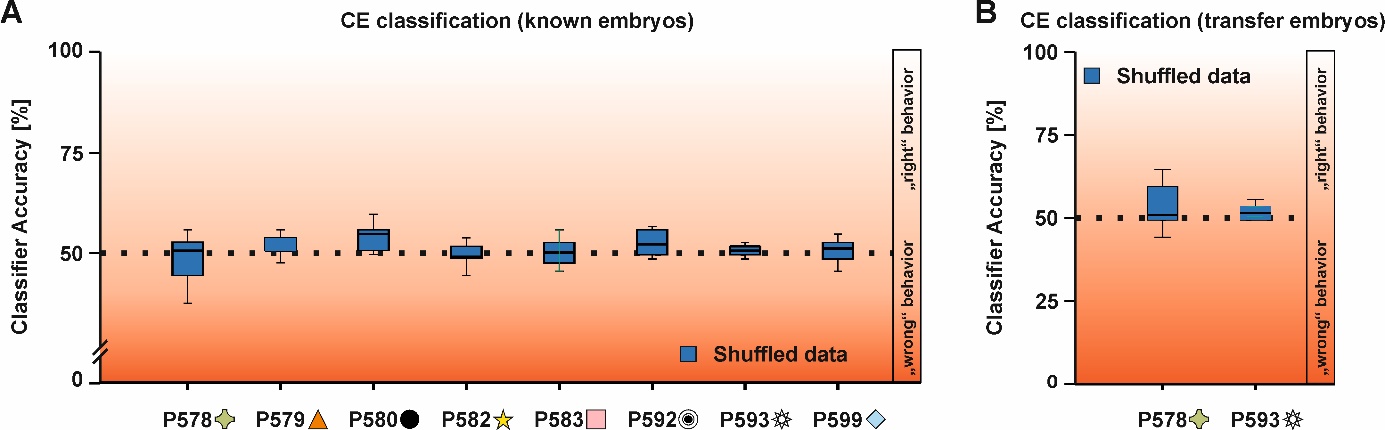


**Supplementary figure 13. Single peck CE (Correct-Error) classification results of the second experimental stage using shuffled input data. A** shows CE classification for the known stimuli. **B** depicts the CE classification for the transfer stimuli. Boxplots represent the lower quartile (Q1), the median and the upper quartile (Q3). Whiskers represent Q1 - 1.5 * IQR and Q3 + 1.5 * IQR.


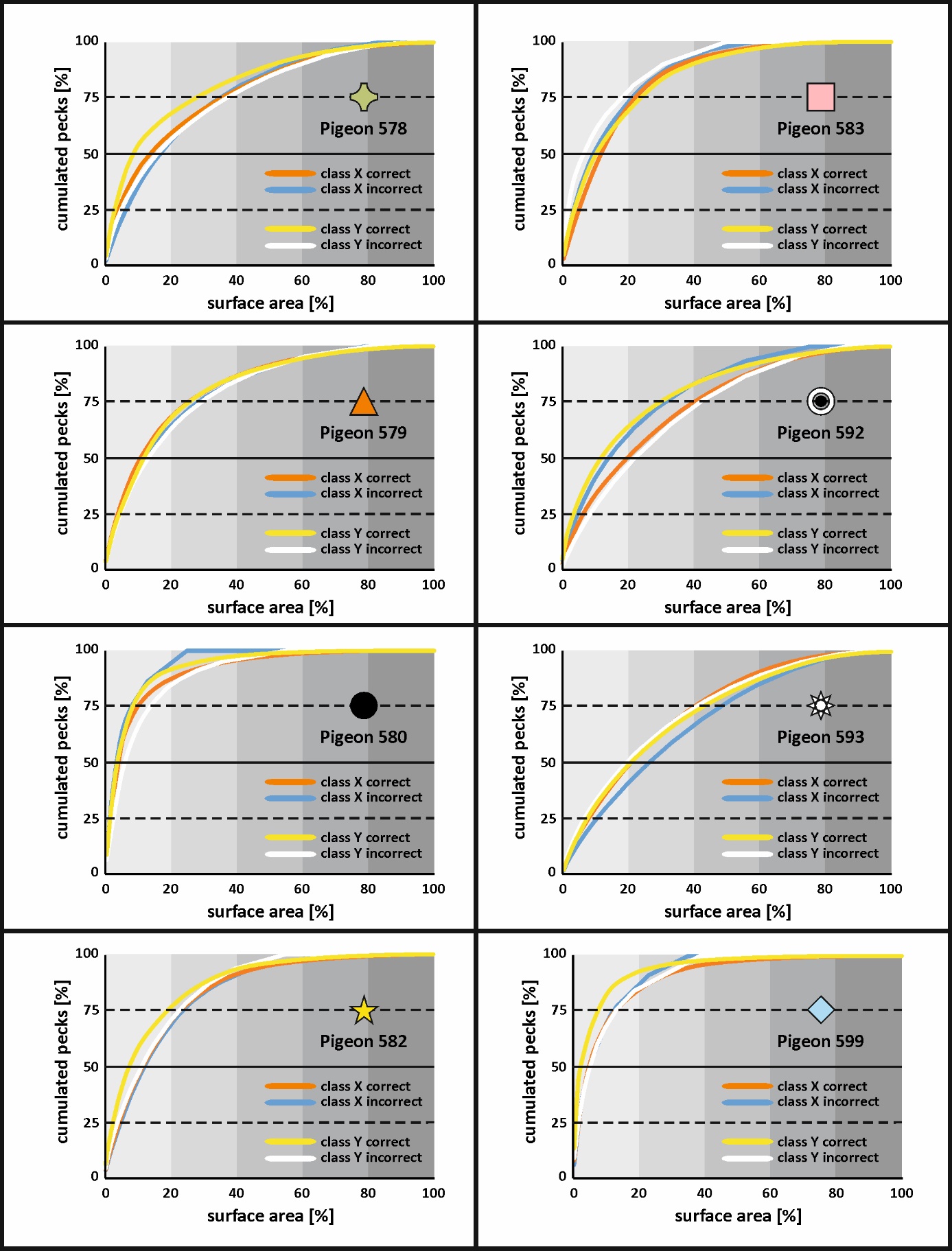


**Supplementary figure 14. Peck distribution analysis.** To analyze the peck distribution we quantified the amount of pecks in each square of the stimulus display that was sectioned into 15 x 15 equally sized squares (see Materials and Methods – Data Analysis). Beginning with the square which was pecked on the most, we computed the cumulated sum by adding the next square in a descending order. Coherent pecking results in a steep increase in the cumulated sum function, since high numbers of pecks are distributed in only a few squares of the stimulus display (e.g. Pigeon 580 and Pigeon 599). Distributed pecking results in a shallow slope of the function, since the overall amount of pecks is distributed over several squares of the stimulus display (e.g. Pigeon 593). Differences in the peck distribution between correct and incorrect responses for the respective stimulus classes can be estimated by the divergence of the cumulated peck functions.


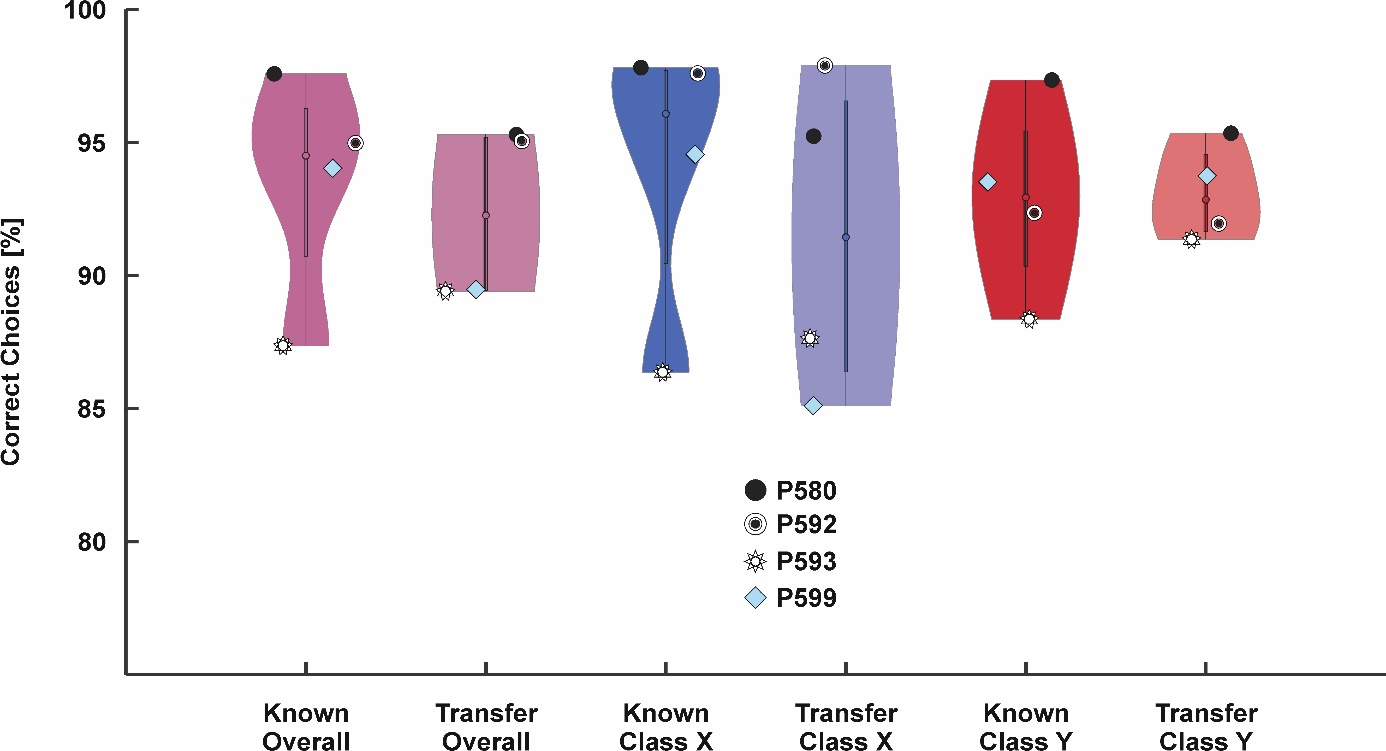


**Supplementary figure 15. Behavioral results.** Violin plot of behavioral performance for known and transfer trials overall and broken down by embryo class. Digital embryos could be categorized in each experimental condition under non-reinforced circumstances. There was no difference between the performance to the known embryos and the transfer to new instances of embryo classes X and Y in any of both conditions. Boxplots represent the lower quartile (Q1), the median and the upper quartile (Q3). Whiskers represent Q1 - 1.5 * IQR and Q3 + 1.5 * IQR.


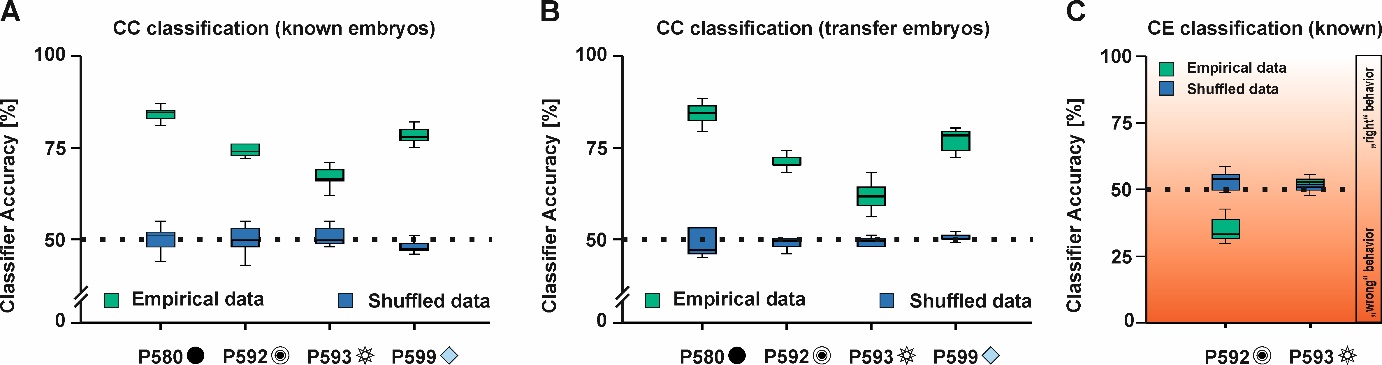


**Supplementary figure 16. CC and CE classification results of the non-reinforced transfer**

Digital embryos could be classified for each animal tested (**A** shows the classifier response for the known stimuli and **B** depicts the classifier results for the transfer stimuli). **C** shows the classifier response for the CE classification. Dark red colors indicate that the pigeons showed the “wrong” behavior for the respective stimulus class and thus a confusion between the categories X and Y. Light red colors indicate that the animals showed the “right” behavior for the respective stimulus class resulting in a confusion within a given category in the CE classification. Boxplots represent the lower quartile (Q1), the median and the upper quartile (Q3). Whiskers represent Q1 - 1.5 * IQR and Q3 + 1.5 * IQR.

**Supplementary table 1. Experimental details for each conducted experimental phase.**


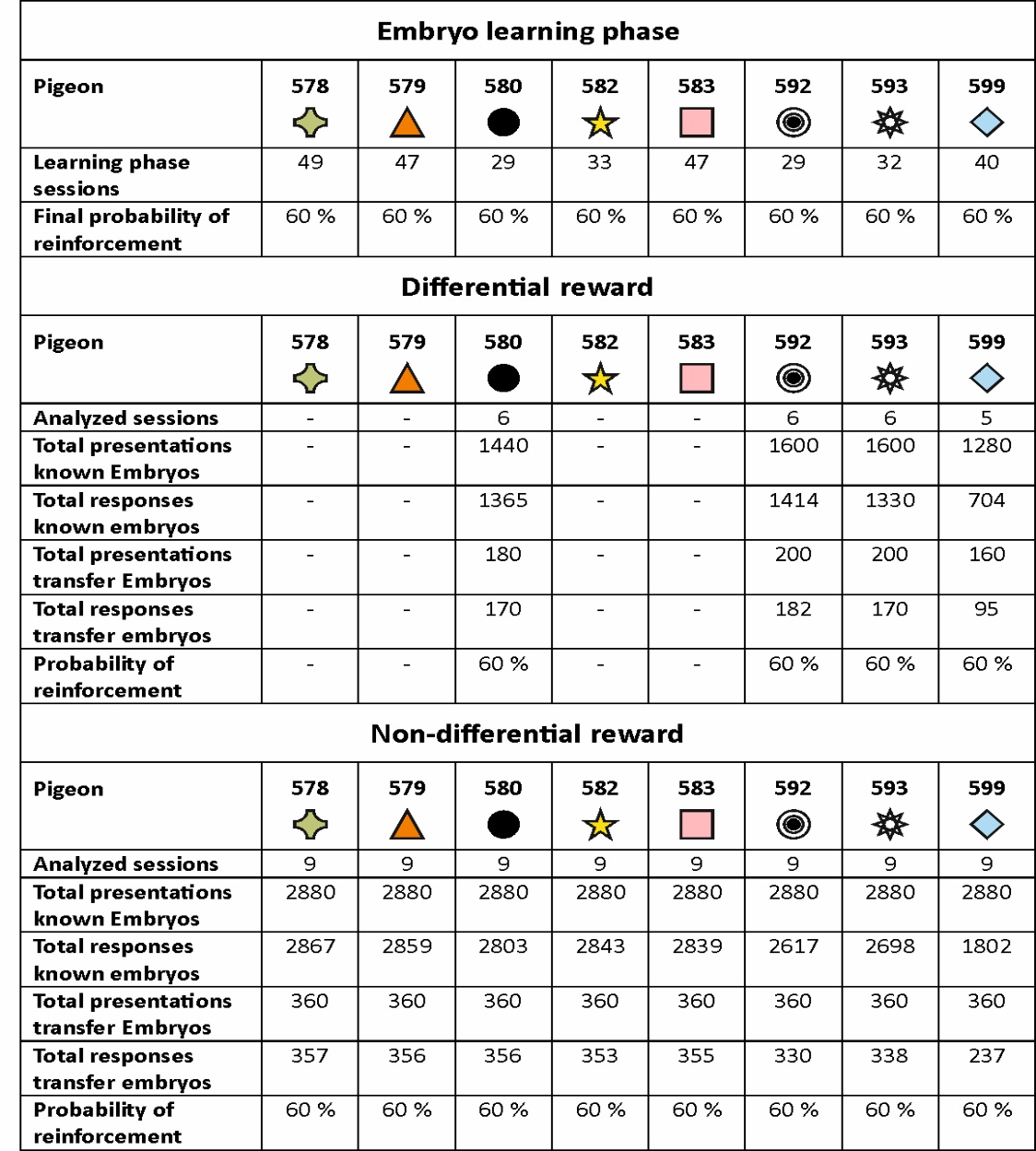


**Supplementary table 2. Comparison of pecking dispersion between correct and error trials for class X and class Y individually for each pigeon using a KS-test.**

| Pigeon | Class X | | Class Y | |
| --- | --- | --- | --- | --- |
|  | Effect size D | p-value | Effect size D | p-value |
| 578 | 0.17 | 0.003 | 0.18 | < 0.001 |
| 579 | 0.2 | < 0.001 | 0.18 | 0.001 |
| 580 | 0.64 | < 0.001 | 0.3 | < 0.001 |
| 582 | 0.28 | < 0.001 | 0.42 | < 0.001 |
| 583 | 0.29 | < 0.001 | 0.4 | < 0.001 |
| 592 | 0.24 | < 0.001 | 0.28 | < 0.001 |
| 593 | 0.16 | 0.004 | 0.09 | 0.322 |
| 599 | 0.54 | < 0.001 | 0.44 | < 0.001 |
